# A Multicriteria Decision-Making Framework for Access Point Selection in Hybrid LiFi/WiFi Networks Using Integrated AHP–VIKOR Technique

**DOI:** 10.3390/s23031312

**Published:** 2023-01-23

**Authors:** Rozin Badeel, Shamala K. Subramaniam, Abdullah Muhammed, Zurina Mohd Hanapi

**Affiliations:** Department of Communication Technology and Network, University Putra Malaysia (UPM), Seri Kembangan 43300, Malaysia

**Keywords:** LiFi, hybrid network, AHP, VIKOR, MCDM

## Abstract

Since light fidelity (LiFi) and wireless fidelity (WiFi) do not interfere with one another, a LiFi/WiFi hybrid network may provide superior performance to existing wireless options. With many users and constant changes, a network can easily become overloaded, leading to slowdowns and fluctuations in data transfer speeds. Access point assignment (APA) is required with the increase of users, which can negatively impact the system performance and quality-of-service (QoS) due to mobility and line-of-sight (LOS) blockage. Many variables could influence the APA process; these variables are considered as criteria, such as the network capacity, the degree of blockage, the speed of the connected user, etc. Unlike conditional APA methods, recent studies have considered treating these variables as “evaluation criteria”. Considering these criteria can offer better and more accurate results, eventually enhancing the APA process and QoS. However, the variety of these criteria, the conflict amongst them, their weights (importance), and priority have not been addressed so far. Moreover, treating the criteria equally might result in inaccurate outcomes. Therefore, to solve this issue, it is essential to investigate the impact of each criterion on the APA process. In this work, a multicriteria decision-making (MCDM) problem is formulated to determine a network-level selection for each user over a period of time The decision problem is modeled as a hierarchy that fragments a problem into a hierarchy of simple and small subproblems, and the selection of the AP network among various alternatives is a considered as an MCDM problem. Based on the previous works, we are not aware of any previous research attempts using MCDM methods in the LiFi research area for network selection. Therefore, this work proposes an access point assignment framework using an MCDM approach for users in a hybrid LiFi/WiFi network. The experiment was conducted based on four phases: Five criteria were identified and evaluated with eleven APs (alternatives). The outcome of this phase was used to build the decision matrix and an MCDM was developed and built based on user mobility and blockages with various scenarios using all the criteria; The analytic hierarchy process (AHP) was employed to identify the criterion of the subjective weights of each criterion and to determine the degree of importance supported by experts’ judgement. Determining the weights in the AHP process considered various investigations, including the consistency ratio (CR) and the AHP consensus indicator, which is calculated using the rank-based maximum likelihood method (RGMM) and Shannon entropy techniques. The VIekriteri-Jumsko KOmpromisno Rangiranje (VIKOR) method is adopted in the selection of the optimal AP for the proper selection of whether a LiFi or WiFi AP must serve the users. The integrated AHP–VIKOR was effective for solving the APA and was the best solution based on using weighted criteria simultaneously. Moreover, the ranking outcomes of the developed integrated AHP–VIKOR approach were evaluated using sensitivity analysis. The result of this work takes the APA for hybrid LiFi networks to a new perspective.

## 1. Introduction

Wireless data has evolved into a resource necessary for our daily lives, and we can no longer operate effectively without it. An annual growth rate of 50% in global data transmission means we are getting closer to a problem called the spectrum crunch [1,2,3,4], a situation where there could not be enough free wireless frequency spectrum to support a growing population of consumption electronics [5]. Therefore, there is increased stress on the network to enhance the user experience. It is projected that the current, already-overburdened wireless fidelity (WiFi) infrastructure would confront an even greater strain due to the limits of the existing radio-frequency (RF)-based wireless technologies and their restricted capacities [6]. One of the newest forms of communication, light fidelity (LiFi), uses visible light rather than radio frequencies to transmit data [7].

The greatest data-transfer rate that can be achieved with this technique is 224 Gbps [8]. Despite its recent history in the research and development arena, LiFi already offers various benefits that might be adopted by businesses throughout the world. There are various ways in which LiFi excels above WiFi: (i) the freedom to use the entire optical spectrum without incurring fees, (ii) the ability to send and receive private messages, and (iii) the ability to work in RF-limited environments.

The downlink propagation medium for LiFi is visible light, making it a novel, high-speed, fully networked wireless transmission system. When used in the uplink, infrared can prevent visible light from interfering with the downlink and keep a room at a constant level of brightness [9]. The downlink connection refers to the data transfer that occurs between a base station (BS) and a user terminal (UE). Of course, there are bounds to LiFi technology, just as there are to every other kind of innovation. When there are obstructions in the way, the LiFi gadget struggles to function properly. In addition, the LOS may be easily blocked by a stationary or moving object of any kind [10], which causes the signal to be lost.

In light of these constraints, several studies have advocated utilizing WiFi and the LiFi system. To address these issues, hybrid LiFi/WiFi networks were set up to combine the fast data-transfer rates of LiFi with the widespread reach of WiFi [6], such as (i) issues with shadows and obstructions [11,12,13,14], in which it is anticipated that the user will no longer be connected to the access point (AP), (ii) data fluctuations, wherein the dynamic setting does not provide constant data rates due to the mobility of users inside the AP coverage region, (iii) several variables such as the optical gain of the reception [9], the robustness of the communication link [15], service accessibility, network capabilities, and the direction of the receiver’s field of vision (FOV) [16,17], etc.

**Figure 1:**
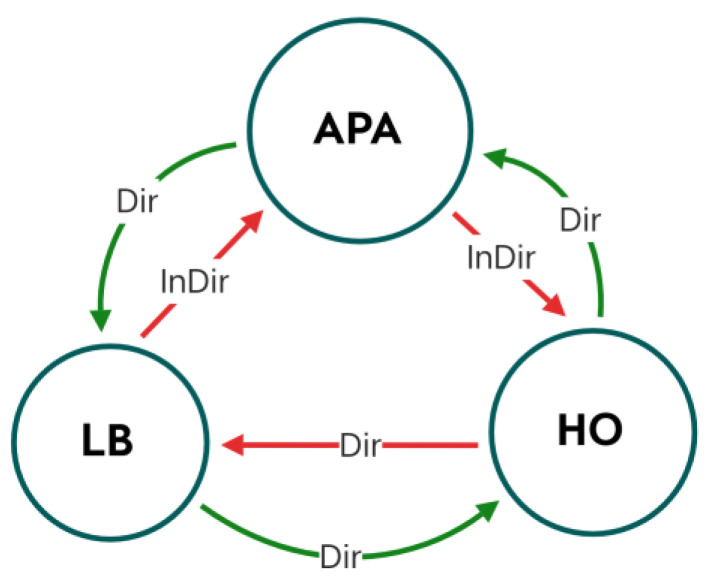
The relationship among LB, APA, and HO.

The aforementioned issues cause complications concerning load balancing (LB), AP assignment (APA), and handover (HO) problems [6,9]. According to the results of prior studies, all three of these issues are linked in some way (see Figure 1). Figure 1 depicts the kind of cause and effect (CE) between the three components (LB, APA, and HO) shown in the figure. Specifically, there are two types of CEs: the first one is called a direct one, while the other one is indirect. The direct CE refers to a variable or a value-related aspect that belongs to a certain factor which could trigger another event. For example, when an HO occurs, it causes a direct need for the APA procedure as well as the LB. On the other hand, when using one of the LB techniques, an indirect effect caused by related aspects (such as data rates, user data-rate requirements, or resource allocation) might take place that could trigger an indirect APA demand depending on the system configurations. When a direct CE happens, a decision must be made for the receiving factor event, whereas when the indirect CE occurs, the CU does not need to take an action or a decision. The LB distributes the network’s workload evenly among all connected devices.

A significant difficulty with the LB arises from the fact that LiFi and WiFi coverage regions completely overlap with one another. LB [18] is made more difficult by factors such as user movement and light-path obstacles, where APA implies selecting the AP from which the user obtains the maximum signal. A LiFi AP typically covers a radius of a couple of meters at most. Therefore, it is inevitable that mobility management concerns would arise, requiring the connected user to be transferred to a different AP, a phenomenon known as horizontal handover (HHO) [14,19,20]. The frequency with which HOs occur increases the chance of connection disruption, which in turn causes packet losses and delays and a poor user impression. However, a VHO occurs between many forms of wireless connectivity. A VHO often needs a lot more time to process than an HHO does, since they use distinct media access control (MAC) protocols. In this hybrid system, a VHO occurs across several wireless access protocols, such as WiFi and LiFi, while an HO refers to switching a wireless connection in progress from one AP to another AP. In most cases, a VHO will significantly prolong a transmission compared to an HHO because of the MAC channels involved [21].

However, most current practices treat these two challenges independently, which can lead to frequent handovers and reduced throughput in a mobile environment with ultra-small cells due to the nontrivial nature of the decision for an HHO or a VHO. The capacity of the network would be severely degraded if VHOs were repeatedly broadcast. It might lead to needless and frequent VHOs, which would reduce throughput. HO methods and algorithms are needed for signal continuity, just as they are for other hybrid networks. According to its mathematical formulation, the VHO algorithm may be developed with any methodology that can be used to fit a single-output, multiple-input function [22].

Unbalanced loads typically emerge when users’ requests for data rates are not consistent throughout geographies, as coverage overlaps across APs are limited in a homogenous network to prevent intercell interference. If such is not the case, then load balancing is unnecessary. Hence, assigning each user to the AP with the strongest received signal strength strategy (SSS) is a common practice in homogenous networks [23]. LiFi and WiFi coverage regions overlap entirely; a WiFi AP has a wider coverage area but less system capacity than a LiFi AP [21]. Therefore, if the SSS technique is used, a WiFi AP would be able to support more users than a LiFi AP.

An integrated AHP–VIKOR is presented in this work to solve the APA problem in hybrid LiFi/WiFi networks, as explained in the next section. Several features must be noticed regarding the solution used in this work, where the advantages of using the AHP–VIKOR as an MCDM approach are as follows: (i) the hierarchical structuring of decision problems; (ii) plausible results; (iii) combining multiple inputs from several persons to a consolidated outcome; (iv) the universality and reduction of subjectivity due to the consideration of the human factor and the verification of data inconsistency; (v) compromising the existing alternatives and solving discrete decision-making on contradictory and noncommensurable criteria; (vi) the unit differences between criteria; vii) finding the closest solution to the overall weight. [24,25]. The disadvantages [24] are: (i) high labor input; (ii) a large amount of initial data; (iii) the limited nature of the assessment scale; (iv) pairwise comparison is a quite artificial way of comparing a set of items; (v) if the consistency index is above 10%, there are problems explaining the request to reconsider inputs; (vi) the VIKOR method is a subjective initial weighting that is challenging to validate.

The thought behind the VIKOR is to choose the alternative that best accomplishes a harmony between two conditions: to be as close as conceivable to the positive–ideal arrangement, and the positive–ideal arrangement speaks to the virtual most-ideal choice that would have been made by choosing the best presentation for each boundary among real propositions. Moreover, the combination of the AHP and VIKOR methods is proposed to cover each one’s disadvantage. The AHP could improve the validity and reliability of weighting in VIKOR. In other words, the hybrid may produce more consistent weighting criteria. In this paper, the AHP and VIKOR will be hybridized for creating a more reliable decision in a manuscript-acceptance system [24,25].

A few interesting facts support our work as motivation, where the priority procedure relies on numerous crucial characteristics when explaining the unique difficulties in ranking APs. Therefore, a multiattribute decision matrix [26,27] describes a user’s choice regarding several qualities in a way that guarantees an appropriate weight is assigned to each attribute, thereby addressing the first problem. As to the second problem, distinct attributes are typically assigned different weights, which further complicates the process.

Finally, the selection of the best AP is heavily impacted by criterion 1 (C1) at a low reference range and other parameters (C2–C8) at a large reference range. The third problem arises because of the trade-off created by the inverse connection between the parameters. Each AP is therefore viewed as a potential solution by the decision-maker, making the ranking process a complicated multiattribute decision-making issue [28]. Given these concerns, the processes pose an essential question of prioritization. To address these problems, a novel approach to intelligent decision-making is required. Moreover, the AHP is a method for ranking alternatives by establishing a hierarchy based on the relative value of goals or qualities [29].

Saaty stated that complex issues needed to be accepted as they were and that the hierarchical examination of complex linkages should be tried, criticizing the inclusion of numerous assumptions in order to simplify complex decision-making processes [30]. Multicriteria decision-making is a strategy that applies the “divide and conquers” mentality to a problem; it has its roots in the field of operations research. In particular, the AHP allows for the comparison of not only quantitative but also qualitative indices, which helps to reduce the likelihood of cognitive mistakes occurring during the decision-making process. As a result, it is put to use in several contexts, such as decision-making (for example, choosing a candidate), resource allocation, dispute resolution, prioritization, and optimization [30].

The term “hierarchy” is used to describe a certain type of system in which the various components are organized into distinct sets based on the entities and characteristics they share. Each grouping is referred to as a “level” [29,30]. To improve upon the current approaches for selecting the optimal AP in a dynamic indoor situation, this research introduces a multi-integration decision-making methodology (AHP and VIKOR). The AHP is useful since it facilitates decisions with several criteria [31]. The AHP is an umbrella measurement theory. It may take either discrete or continuous paired comparisons and utilize the results to create ratio scales. The AHP, in its broadest sense, is a nonlinear framework for combining deductive and inductive reasoning outside of the syllogistic method by weighing several aspects simultaneously, permitting reliance and feedback, and making numerical trade-offs to reach a synthesis or conclusion [32]. The contribution of this work is summarized as follows:

For the first time in the LiFi research area, we propose an access point assignment framework using an MCDM approach including an integrated AHP–VIKOR approach. To the best of our knowledge, no similar study has presented the following aspects summarizing the contributions of this work. An MCDM problem is formulated for the presented hybrid LiFi/WiFi network.

1. We identify and determine the criterion weights using the AHP.
2. The values of the criteria weights are examined by various investigations, including CR and AHP consensus indicators. Then, the AHP consensus is calculated using the RGMM and Shannon entropy techniques.
3. A multicriteria decision matrix is developed and built based on user mobility and blockages with various scenarios using all the criteria.
4. We prioritize the selection of alternatives (APs) using the VIKOR to accurately select the optimal AP in the hybrid LiFi/WiFi network.
5. The robustness of the integrated AHP–VIKOR approach was evaluated using sensitivity analysis.

The rest of the paper is organized as follows: Section 2 shows the related works of the study, including the research gaps. Afterwards, Section 3 presents the methodology, including the integrated AHP–VIKOR phases. Section 4 indicates the results and discussion. Finally, the paper is concluded in Section 5.

## 2. Related Works and Research Gap

**Figure 2:**
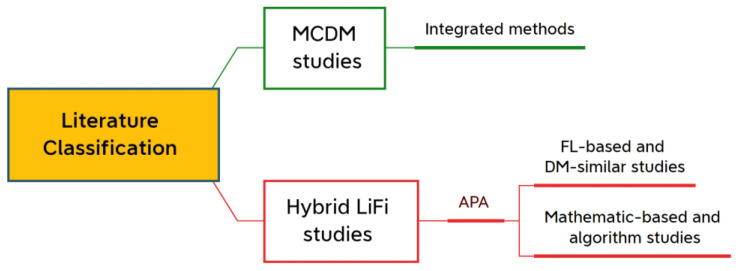
Literature review classification.

This section discusses the related works, including the related MCDM studies and all hybrid LiFi networks considered in the AP assignment, AP selection, HO management, HO skipping, and LB. Studies are divided into two categories; the first will focus on integrated MCDM studies. On the other hand, the second group of articles will consist of LiFi-related studies, and those will be divided into two parts: the first part includes all standard schemes and algorithms wherein the models and methods utilize mathematics and/or equations to reach the intended objective. The second part includes the studies of methods with the principle of the DM approach, most of which used fuzzy logic (FL) methods. Figure 2 shows the classification of the studies.

In this section, the related studies which focus on using integrated studies will be discussed. The VIKOR method was developed as an MCDM approach which determines a compromise solution which is acceptable for all decision-makers and solves a discrete multicriteria decision problem.

### 2.1. MCDM Studies

Integrated MCDM studies were used in different aspects and different environments, where the evaluation of the satisfaction level of citizens in municipality services was conducted [33], and where, in [34], a selection of the most efficient procedures for the rectification of the optical sight of the long-range rifle was introduced. Moreover, the AHP–VIKOR was used for self-driving vehicles [35], location-selection problems [35], telemedicine and health environments [36,37,38], industry and product development and costume satisfaction [39,40,41], strategies selection [42], prioritizing the energy source [43], agriculture [44], and risk assessment [45]. In addition, MCDM techniques were used in network and wireless communications.

The MCDM integrated methods, such as AHP–VIKOR, were used for network selection in the heterogeneous network environment. Specifically, the objective in [46] was to describe the applications of three MCDM methods for solving the network selection problem. The fuzzy analytic hierarchy process (FAHP), the FAHP technique for order preference by similarity to ideal solution (TOPSIS), and the FAHP preference ranking organization method for enrichment evaluation (PROMETHEE) were proposed to choose the best network among the various network alternatives. Solving the issue of the selection of the most appropriate network in a heterogeneous wireless environment is one of the critical issues to provide the best quality-of-service (QoS) to the users.

The study by [47] described a novel MCDM method to evaluate and select the suitable network for a wireless network environment. Their proposed technique involved an FAHP that was integrated with a technique for order preference by similarity to the ideal solution (TOPSIS) and VIKOR. The FAHP was used to determine the criteria weights, whereas the TOPSIS and VIKOR were used to find the performance ranking of the alternative networks. The work focused on five network alternatives, WLAN, GPRS, UMTS, WIMAX, and CDMA, with ten evaluation criteria, including bandwidth, latency, jitter, BER, retransmission, packet loss, through put, preference, security, and cost.

In [48], a vertical handoff mechanism was deployed where an active call or session was transferred from one access point to another depending on the services required by the end user. In order to provide ubiquitous access to end users in such an environment, the optimal network selection among a large number of networks available required consideration of multiple criteria such as cost, delay, jitter, and available bandwidth; moreover, their motive behind this was to check the effectiveness of normalization method used in the original VIKOR method. Therefore, the multiattribute decision-making (MADM) method analyzed the performance of the VIKOR MADM method for the two most popularly used weighting methods, AHP and ANP, along with three commonly used normalized methods.

In addition, in another study [49], the performance of the VIKOR MADM method for vertical handoffs in heterogeneous networks with various weighting methods was studied, wherein the authors proposed the V-ANP, which resulted in fewer handovers and ranking abnormalities; moreover, the performance of a new network selection strategy proposed by the authors was compared with different weighting methods such as subjective weighting and the traditional AHP method. During the handoff, all of these criteria should be kept in view for network selection where these include handovers and ranking abnormalities as well. These are the major issues for any MADM method. Although the risk of the M method, as well as the risk of ranking abnormality, is slightly more than the V ranking, and the abnormality is slightly more than for the V-AHP for three traffic classes, network selection is appropriate for them. Furthermore, the authors in [50] proposed the VIKOR as one of the MADM methods in the choice of the next-handed network in the heterogeneous wireless network and they used the AHP to produce the weights used by the VIKOR to consider multiple criteria in the process of ranking the available networks, where the VIKOR boosted the QoS. Their aim was to improve the delay in the packet-loss rate in interactive and background applications and to reduce the delay significantly in the streaming class against the SAW, TOPSIS, and MEW.

The AHP and interval VIKOR methods were used for selection in integrated VANET and 3G heterogeneous wireless networks [51], where selecting the optimum network and gateway was the key point in the phase of the vertical handover decision-making process. They aimed to improve system efficiency by following the objectives of the defined heterogeneous network. In the proposed method, the AHP method was used to determine the weights of the criteria, and the modified VIKOR, which meant a multicriteria optimization and compromise solution with pronunciation, whereby the VIKOR method was used for deciding the heterogeneous networks. In [52], a combined application of the VLC and Femto and a multiattribute VHO decision-making algorithm was introduced. The integration between the AHP and CG (cooperative game) was proposed, where the AHP was utilized to deal with the importance comparisons of multiple criteria according to different traffic types. The combination of both methods evaluated the candidate decisions’ abilities to handle VLC signal blockage or overload.

### 2.2. LiFi Studies

#### 2.2.1. The First Group (Mathematics-Based and Algorithm-Based Studies)

The studies of [11,53,54,55] and [56] considered the LB, whereas in [53], the authors described an approach for cooperative load balancing in a WiFi/VLC hybrid network to achieve proportional fairness. Through the analysis of the LB issue of many VLC APs and a WiFi AP, this approach was designed using the proportional fairness (PF) measurement of the users as its foundation. In addition to describing a dynamic LB scheme, the paper [54] also discussed a utility function that took into account both system throughput and fairness. This study delved into several questions about the proposed LB scheme in a hybrid LiFi/WiFi network, including how the handover affected the LB, the coverage areas provided by the various APs, and the throughput that can be obtained by each AP.

In [55], the authors provided two variants of the same optimization methodology—the Assign WiGig First SOA (AWFS) algorithm and the Concurrent Assign WiGig First SOA (CAWFS) method (SOA). Since their goal was to deliver the maximum possible data rate, the LB was considered; in this case, only users requiring the lowest possible LiFi data rate would be assigned to the WiGig AP, while the remainder of users would be assigned to LiFi APs. Using two scenarios for hybrid LiFi/WiFi networks, the study by [11] analyzed the QoS performance of wireless networks with QoS-driven load balancing: single-AP association (SA) and multi-AP association (MA).

The LB issue has been fixed for both single-AP and multiple-AP setups. Furthermore, QoS-driven load balancing and throughput-driven load balancing were contrasted by analyzing the quality-of-service performance with varying packet arrival rates and user loads. The goal was to reduce the packet-loss ratio and delay. The authors of [56] detailed a user-focused implementation of LiFi CoMP-JT clustering. The LB architecture solved the concomitant issues of unequal cell loads and LiFi ICI in WiFi/LiFi networks.

The design took into account how the LiFi CoMP-JT clustering and LB would affect performance. The goal was to increase system throughput while keeping fairness proportionate. The research by [20,57], on the other hand, looked at APA issues and HO skipping methods. The study by [57] proposed an HO-bypassing algorithm based on the received reference signal strength (RSRP). The new technique incorporated the RSRP value and its rate of change to calculate the HO target and whether to skip an AP. This novel method can greatly reduce the handover rate, since it takes into account the user’s velocity, which affects the rate of change in RSRP.

In [20], a one-of-a-kind HO approach for deciding whether or not to skip an AP was proposed. This method incorporates both the RSRP and its rate of change into its assessment. Skipping HO tactics refer to these methods. Furthermore, it provided an adaptive WiFi range that follows the user’s pace, allowing a mobile user to stay connected to WiFi and prevent repeated HOs. The goal was to get the HO rate down as much as was feasible.

On the other hand, the studies by [14] and [9] addressed the APA and HO issues, respectively. In [14], a novel HO mechanism based on machine learning was introduced for hybrid LiFi and WiFi networks. This technique uses a dynamic coefficient to adapt the choice between LiFi and WiFi based on channel quality, resource availability, and user mobility. This technique uses a dynamic coefficient to adapt the choice between LiFi and WiFi based on channel quality, resource availability, and user mobility. An artificial neural network (ANN) was used to train data for a variety of use-cases, including user speed, LiFi AP distance, LiFi AP height, and the number of LiFi APs relative to WiFi APs. To verify the ANN model and evaluate the performance of the proposed handover strategy, simulations were undertaken.

In [9], the authors focused on APA and HO problems with user mobility and user density. For high data rates per user and consistent connections with fewer HOs, the authors presented the innovative three-phase handover management and AP transition (TPHM-APT). Their plan emphasized decreasing the total number of HOs [58] to boost dependability while keeping the number of users per LiFi node relatively low.

#### 2.2.2. A second Group (FL-Based and DM-Similar Studies)

This collection represents the most related studies to our work, which are [21,59,60,61,62,63]. They all worked on the APA using the FL method similar to the DM method for APA in different hybrid networks. An FL-based dynamic load balancing technique was proposed in [59] to lessen the effects of handovers in mixed LiFi/RF deployments. Users who are in a constant state of motion or who are suffering a temporary shadowing effect will be better served by the suggested method than by the APs chosen by the default algorithm thanks to the utilization of information on user speed and the temporal average of the LiFi SINR.

In addition, the authors of [60] considered homogeneous and heterogeneous networks, as well as the APS. In addition, a two-stage APS technique was proposed for LiFi/WiFi hybrid networks. First, they developed a fuzzy logic approach to decide who should be given access to the WiFi network. Second, the remaining users were placed on a LiFi network to maximize throughput and simultaneously decrease system complexity. Additionally, to better serve end users, a hybrid networking architecture combining OCC and LiFi was proposed in [61]. User assignments in the hybrid LIFI/OCC networks were made using fuzzy logic. If the following FL principle is applied, it could achieve optimality while drastically lowering the computational cost.

Another study by [21] proposed a joint optimization problem to determine a network-level choice for each user over time. Load balancing is made more difficult by factors such as user mobility and light-path obstructions, which may be avoided with the use of the novel FL-based technique described here. Moreover, in [62], we see research towards a handover decision procedure for a combined LiFi and WiFi network. ANN and fuzzy logic (FL) HO selection algorithms were introduced to identify which access points (APs) should be used for a given user and whether or not to suggest an HO depending on the user’s velocity.

Access point selection (APS) in downlink scenarios may be challenging because of the plethora of bands. In [63], as in the previous study, FL was used in the APA scheme to determine if a user would connect to WiFi APs, LiFi APs, or mmWave APs. As a result, a two-stage procedure was introduced to effectively associate user equipment (UE) with network APs. In the beginning, each UE picked its favorite AP on each network (mmWave, WiFi, and LiFi) individually. Next, the assignment of each UE to a network was performed by an FL algorithm.

Table 1 provides a synopsis of all the above-searched studies, outlining the most important points. Overall, the table shows that the FL-based DM approaches were addressed in the majority of the papers that were included in the analysis. There are three stages to an FL system: fuzzification, rule assessment, and defuzzification [64]. The majority of the aforementioned research suggested employing FL to simplify operations. In the first stage, for instance, membership functions are used to map single-valued parameters onto the values of a fuzzy set. This is often done in MATLAB using a collection of membership functions (MFs), which map each parameter onto one of three levels of significance (low, medium, and high). Step two involves creating fuzzy constraints to assess the pros and cons of a potential access type for a network or assignment option. These principles are intuitively determined and obvious; hence, no methodical process was followed in weighing the various elements. With so many criteria to consider, such as WiFi throughput, LiFi CDT, etc., all of these standards were given the same priority. WiFi, for instance, is best suited for users who are always on the go (rule 1), whereas LiFi/Wi-Fi is preferred by those who move at a slower pace and who experience frequent light-path obstructions (rule 3). Two potential values were similarly used in the process’ input and output. After that, a yes/no or positive/negative value was determined for each potential participant.

Another significant limitation which impacted the output of decisions was found in the studies by [21,60], which consisted of six rules in the fuzzy rules, two outputs of all rules were not specific, and that formed 33.33% of the options, which were not accurate and were confusing in the APA process for the hybrid LiFi network. For instance, the output values of the ruleset in the study by [21] were 0.6, 0.2, and 0.3 for ‘LiFi only’, ‘WiFi only’, and ‘LiFi/WiFi’, respectively. That means the output ‘LiFi/WiFi’ considers the hybrid network in general without determination. Moreover, standard FL techniques that produce a direct solution may not produce a suitable outcome despite being well-designed.

Based on our literature analysis, it became evident to us that the use of the MCDM as a decision support tool has been used in a variety of applications and domains. Wireless technologies and communications were some of such areas where many MCDM techniques were applied. At the same time, the MCDM varied in how it was applied, where some utilized a singular MCDM approach, and some utilized two or more integrated MCDM methods to propose a proper solution for their complex problems. Amongst the most common and well-known utilizations were those utilizing the AHP–VIKOR, where the former weights the criteria and the latter ranks the alternatives for a proper selection.

Motivated by this, this integration was studied and investigated, and the literature shows it has been used for some wireless technologies and communications cases, but not with the case study of this research. Therefore, it has been used and we are unaware of any previous attempt to use the AHP–VIKOR for the problem of the APA in hybrid LiFi/WiFi networks, which is the main focus of this research. The MCDM is an expansion of decision theory that accounts for decisions with multiple objectives [65,66,67,68,69]: “an umbrella to represent a range of formal procedures that explicitly evaluate numerous criteria in helping persons or groups to explore important options”, as defined by the research of [70]. The primary goal of the MCDM is to make a call by evaluating potential solutions according to several criteria [28,71]. As a result, the APA in hybrid LiFi networks can benefit from the usage of the MCDM and achieve greater accuracy because:

1. First, it considers the relative importance of each criterion, which might otherwise lead to erroneous conclusions and larger error rates if ignored.
2. Second, it involves a decision-making process that prioritizes the available options. In this circumstance, APs are the alternatives. The output value might be impacted if priority is ignored at the last step of AP selection.
3. Finally, it considers the margin of error.

From the aforementioned research, we learn that no studies have separated the effects of individual variables and those of multiple variables on the MCDM method. Keep in mind that if you decide to use a method that requires several evaluation criteria and qualities, the difficulty level may rise. Therefore, knowing the precise situation for all values according to all criteria and their weights is crucial. Given the complexity of a dynamic and hybrid wireless network, characteristics must be prioritized for the effective coordination and administration of the APA.

## 3. Methodology

**Figure 3:**
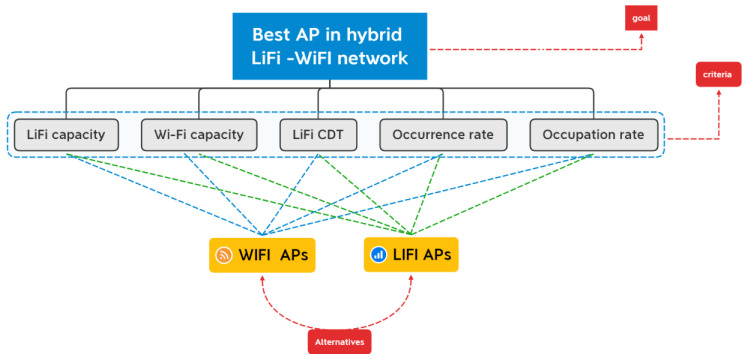
The analytic hierarchy process (AHP) diagram in hybrid WiFi/LiFi networks.

The development methodology of the proposed prioritization framework is introduced and discussed in this section. The system model of an indoor hybrid LiFi network, which consists of one WiFi AP and several LiFi APs, is proposed and shown in Figure 3. The main evaluation criteria and the alternatives used in this study as a system model are shown in Figure 3.

The WiFi AP is installed in the room’s geographic center, providing coverage to all four corners. Each LiFi AP has a limited coverage region and is built within an LED light fixture in the ceiling. By reusing the optical spectrum, the LiFi APs significantly reduce the amount of interference between cells. Specifically, white light may be generated by combining the outputs of many-colored LEDs, and each color can be adjusted independently. At the receiver, optical equipment, such as that shown in [72], can separate these colors of light. Time-division multiple accessing (TDMA) can be used to enable one AP to serve multiple users, whereas each user can only be assigned to one AP [21]. Different spectral ranges used by the LiFi APs ensure that their operations are mutually independent. Random waypoint (RWP) is assumed for mobile users [9]. Users are supposed to proceed in a straight route between randomly placed waypoints. User velocity is a uniformly distributed random variable between 0 and the maximum velocity. The PDs of all users face upward, and users can only link to a single AP. During this time, an AP is dynamic and agnostic.

Let α denote the type of network, which is the LiFi and WiFi termed as ‘LiFi/WiFi’. Let *k* denote the type of network access which falls into two options: ‘LiFi’ or ‘WiFi’. Therefore, we focus on deciding the type of network access. Specifically, let *X_k__,__u_* = 1, meaning that user *u* chooses a *k*-type of network access, while *X_k__,__u_* = 0 means otherwise; $p_{u}^{a}$ denotes the proportion of time that the α-type network allocates to user *u.* The AHP requires stating the aim and identifying possible alternatives. Since there are typically numerous decision-making criteria, the next stage in the AHP is to create a hierarchy with the most general criterion at the top.

Next, the AHP calculates the weight of each criterion in relation to the other criteria with which it is linked (i.e., establishing the weights for each criterion). At last, the AHP compares each option to every other option based on the criteria at the very bottom of the criteria pyramid.

The result will be a ranking of options that meet the stated aim based on the relative importance of each criterion. The AHP process looks like this: the issue is defined and the solution is developed.

1. By breaking down the traits needed to reach the target, the criteria and/or subcriteria may be determined.
2. The criteria and subcriteria serve as the building blocks for the hierarchy, which rises from the lowest to the highest levels. The next step is to develop a comparison matrix between the various criteria. In addition, weights are calculated by comparing two criteria according to relative preferences of 1–9; when the number of alternatives is 𝑛, a total of 𝑛(𝑛 − 1)/2 comparisons are made
3. Finally, the consistency ratio (CR) is determined to ensure that answers are fair. Priority vectors are used to calculate the CR value, and as Saaty underlined, only when the consistency ratio is 0.10 or below [29,30,73,74].
4. The AHP presupposes the four points below. The first example is a reversal. The value of preference should fulfill the reciprocal requirement when the two elements are coupled and compared.

**Figure 4:**
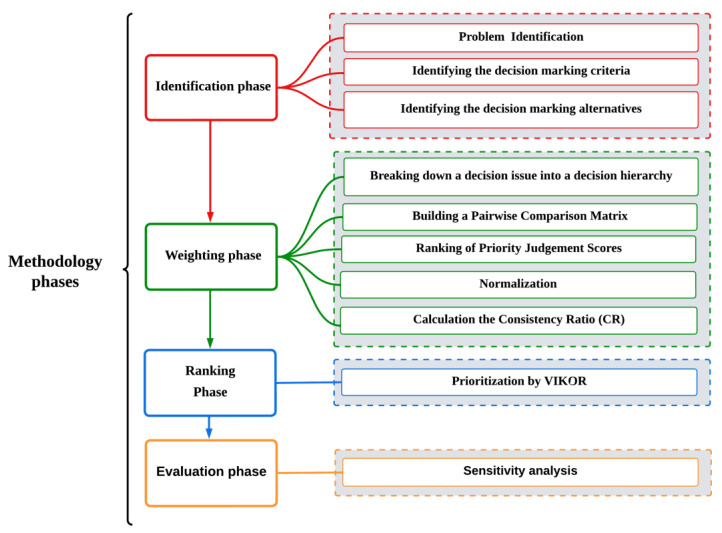
Methodology phases of AHP–VIKOR.

For instance, if 𝐴 is 𝑥 times as important as 𝐵, 𝐵 is 1/𝑥 times as important as 𝐴 or vice versa. The second is homogeneity. A scale with a finite range is used to illustrate the importance. Thirdly, we have dependence. Each level’s components must rely on its superiors. Expectations are the fourth and final assumption. This presumes the appropriate level fully incorporates the reasons for making decisions [29,30]. All the phases and steps of the AHP–VIKOR methodology are divided into four phases: (i) the identification phase, (ii) the weighting phase, (iii) the ranking phase, and finally (iv) the evaluation phase. All the phases and their steps are illustrated in Figure 4.

### 3.1. Identification Phase

This section discusses the following: (I) problem identification of the APA and HO, (II) criteria identification, and (III) identification of the alternative.

#### 3.1.1. Problem Identification

In this section, the actual problem definition is explained to figure out what the actual issue is that needs fixing. According to the suggested procedure, either “LiFi” or “WiFi” network access will be identified. Those two distinct groups are the only ones allowed on a specified network. These subscribers are transferred to a different source (VHO or HHO) within the same network if necessary. The next part formulates a centralized optimization problem using the categories. Based on these classifications, a centralized optimization problem is created. Both factors add complexity to the APA/HO problem: (i) In contrast to LiFi Aps, WiFi Aps have a smaller coverage area but a higher system capacity, and (ii) the service regions of the two networks may overlap.

The purpose of this study is to complete the criterion weight and value hierarchies used in AHP pairwise comparisons, as seen in Figure 4. However, the proposed method works to prevent ping-pong (PP) effects [75]. When a user equipment (UE) time-of-stay within a small cell is shorter than the threshold, and the UE subsequently HO to another AP, the resulting HO is deemed superfluous [76]. The PP impact happens when a user’s call is transferred back to the original cell within the crucial period after the HO decision was made at the neighboring cell. The PP rate is the number of PP HOs divided by the sum of all HOs (including those that did not involve PPs), the number of successful HOs, and the total number of HOs.

#### 3.1.2. Identifying the Decision-Making Criteria

In this step, the required evaluation criteria are identified and explained to choose the alternatives. The data should be written as C = [*Cj*], where j = 1 and N represents the number of criteria. The number of criteria used in this study is five, including the WiFi capacity, LiFi capacity, LiFi CDT, occurrence rate, and occupation rate.

#### 3.1.3. Definitions

Cell dwell time (CDT) is the typical amount of time a mobile device stays at the same access point (AP) before an HO occurs. A user’s CDT may change over time based on factors such as the user’s speed and the direction in which the user is traveling. The study by [18] considered user mobility and utilized the CDT value to determine how much of the time the HHO would use relative to an α-type network, all while using the HHO’s overhead. In other terms, the CDT, also known as the HO dwell time, is “the time interval the mobile terminal (MT) can spend interacting with the cell it is departing (current cell) until the channel quality reaches a minimal threshold,” as stated in [77].

The quality of the channel is expected to be dependent on the strength of the signal the MT gets from the current base station (BS) during intercell HOs.

It makes sense to solely consider the impacts of shadowing and ignore the rapid fading effects. The crossing events caused by fast fading dictate short fading intervals; therefore, they may be disregarded when trying to identify the requirements for active call terminations. The HO dwell-time behavior depends on the terminal speed (module and direction) and the HO residual margin *M_ho_*, which is measured with decibels (dB) and can be given by two variables, the HO threshold *Q_ho_* and the quality threshold *Q_th_*, which represent signal power levels [77]. If the *M_ho_* is sufficiently large, then the *Q_ho_* and *Q_th_* are sufficiently spaced. *R*_1_ is the straight-line distance between the current BS and the location from which the MT is requesting an HO, and this distance is equivalent to the down crossing of the *H_o_* as measured by the received power. However, the distance that corresponds to the *T* is symbolized by the symbol *R_2_*. The residual margin is proportional to the ratio of *R*_1_ minus *R_2_*, or the depth of the HO region along the cell radius.

The findings of the study by [77] stated that, under quite general assumptions, it is seen that the CDT tends to be Gaussian distributed for high values of the *M_ho_*, i.e., for a high percentage of overlapping areas. However, low values of the CDT become very likely when the *M_ho_* is quite low.

Another finding of the study stated that the practical insights of the paper can be focused on an efficient design of handover procedures, which accounts for both the initiation and the execution phases. In particular, if the *M_ho_* is high enough, queue-based prioritization schemes could benefit quite long waiting times. High-capacity communication systems are in high demand as data traffic continues to increase at an alarming rate. Because of this, a spectrum crunch is imminent for RF-based wireless communication [78]. In contrast, the optical domain offers a vast, unlicensed bandwidth that is immune to electromagnetic interference and comes with built-in privacy protections. In order to allow a larger capacity distribution of connection between LiFi access points dispersed along the interior environment using OFDM, an effective backbone network must be designed [79].

In comparison to current technologies, the primary goal of the new standard is to increase capacity and transmission performances by a factor of one thousand. In order to boost data speeds, it is necessary to think about more space in the electromagnetic spectrum and to use a wide variety of novel internetworking strategies. The network’s spectral efficiency and capacity will increase as the communication needs of users on the same frequency reuse the available frequency to the greatest possible extent [80]. Hybrid networks can increase capacity and reliability when the physical layer configurations are kept the same [56].

According to the research by [81], the requirement for tiny cells does not pose a problem from a system-capacity standpoint. This is due to the fact that optimizing system performance in modern cellular communications relies heavily on the reduction of cell sizes. Accordingly, contrary to common belief, terrestrial transmission at higher frequencies is now a viable alternative. However, as cell sizes continue to shrink, it becomes increasingly difficult to build the necessary infrastructure to sustain them.

Providing a high-tech backhaul network is one such instance. According to the study by [81], there are substantial advantages to using a hybrid LiFi/WiFi system in terms of capacity, resilience, security, and dependability. This bolsters the argument that LiFi, when viewed as a supplementary wireless networking approach, may not only supply more free and huge wireless capacity, but also help to improve the spectrum efficiency of already-existing RF networks. Pre- and postequalization, high-order modulation using orthogonal frequency division multiplexing (OFDM), and other methods, have been investigated to optimize the modulation bandwidth and obtain large data speeds in the region of several Gb/s utilizing a single LED [82,83].

LED modulation bandwidth has been shown to grow with increasing bias current up to the point of saturation [84,85]. LEDs, however, are nonlinear devices. That is, there is some nonlinearity in the relationship between the driving current and the optical power output. In [86], the best DC bias for a LiFi network was determined; this bias allows for maximum throughput by expanding the modulation bandwidth. Evidence suggests that an LED’s modulation bandwidth expands from its linear midpoint toward its DC bias.

The authors then analyzed how these changes the received signal-to-noise ratio (SNR) and reported on two methods for determining the best DC bias point to maximize the link’s capacity. Results from experiments demonstrate that transmission rates may be improved by as much as 36% just by raising the bias current from the linear region’s midpoint of 20 mA to the optimal bias current of 30 mA. The same procedure allows the methods to be applied separately on user-end devices. The experimental findings also show that the suggested approach in [87] may significantly increase the LiFi channel capacity of a wireless LiFi transmission link based on a white LED.

#### 3.1.4. Analysis

The decision matrix must be constructed after the criteria have been identified and discussed. To do this, values must be determined for each criterion. It is crucial to understand the origins and rationale behind the values. This is because some criteria have been identified in previous studies, while some other criteria were never given a specific value. To solve this issue, we will make calculations and measurements either through simulation or based on the analysis of previous models in the literature. For network capacity including WiFi and LiFi, many factors contribute to the final value of the network capacity for the user. First of all, the capacity of the network starts with the size of the spectrum where the size of the infrared and visible light spectrum together is approximately 2600 times the size of the entire radio frequency spectrum of 300 GHz [88]. The RF spectrum ranges from 0 to 3 ×10^10^, while the LiFi spectrum (VL) ranges from 4 ×10^14^ to 7.9 ×10^14^, which makes it 2600 times bigger. The throughput and data transmission rates were also thought to be crucial components that exemplify the notion of capacity [89,90]. Moreover, the network capacity is significantly impacted by the type of modulation and data transmission technique [79,88,91]. Contrarily, the increased number of APs has an influence on system capacity but the cell size (coverage area) has no effect [81]. Another element in this situation is user data consumption, which is also taken into account when integrating with other networks such as 5G [92], which includes extra tiers such as small cells (SCs) [90]. The network’s downlink capacity diminishes as distance and peak and average power limitations increase [93]. Other elements could be added, such as physical layer setup [56], network infrastructure [79,92], hardware and software design [94], and others. The link between the AP and the user’s capacity can be calculated using the Shannon capacity for WiFi as well as the signal-to-interference-plus-noise ratio (SINR) of the WiFi channel while taking into account bandwidth, the WiFi AP’s transmitted power, the WiFi channel gain, and the power spectral density (PSD) of noise at the receiver [20].

**Figure 5:**
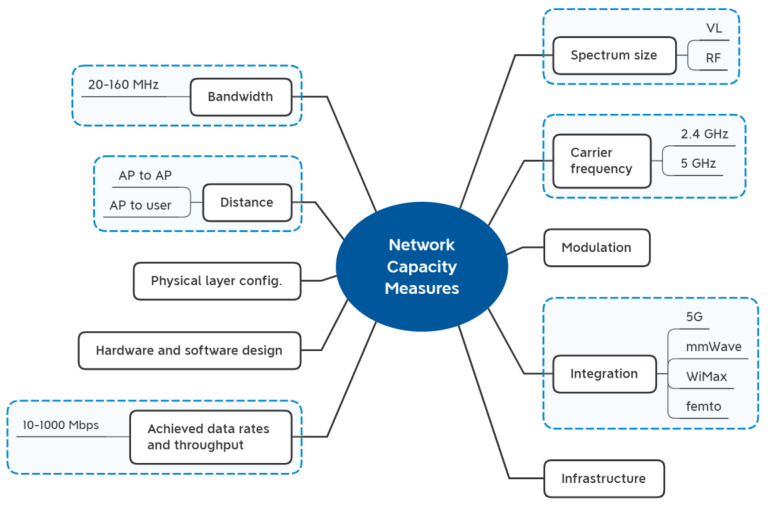
Factors involved in measurements of the system’s capacities.

Additionally, the electrical SINR for non-negative signals, the bandwidth of the LiFi, the SINR of the LiFi connection, the PSD, the detector responsivity, the average modulated optical power, and the total gain of the LiFi channel can all be taken into account when calculating the LiFi capacity [20]. Figure 5 summarizes the factors as explained above. According to [21], the average capacity that the AP can provide to the user was formulated based on the Shannon capacity [53], as follows:

(1) $$r_{i,u}^{\left(t\right)}=\left\{\begin{array}{c} \frac{B_{i}}{2}\log_{2}\left(1+\frac{e}{2\pi }\gamma_{i,u}^{\left(t\right)}\right),\;for\;LiFi\\ B_{i}\log_{2}\left(1+\gamma_{i,u}^{\left(t\right)}\right),\;for\;WiFi\; \end{array}\right\}$$

where $r_{i,u}^{\left(t\right)}$ is the capacity that AP *i* can provide to user *u*, $B_{i}$ is the system bandwidth of AP *i*, and $\gamma_{i,u}^{\left(t\right)}$ denotes the received signal-to-noise ratio (SNR) regarding the link between AP *i* and user *u* at time point *t*. On the other hand, the calculations of the average capacity for the α-type network took into account the HO overhead and the number of users for each network in addition to the same variables in (1), using the formula:

(2) $$r_{u}^{\propto }=\left\{\begin{array}{c} \frac{1}{T}\sum_{i\in \propto }^{n}Xi,u\;\int_{0}^{T}\frac{B_{i}}{2}\log_{2}\left(1+\frac{e}{2\pi }\gamma_{i,u}^{\left(t\right)}\right)dt\;,\;for\;LiFi\\ \frac{1}{T}\sum_{i\in \propto }^{n}Xi,u\;\int_{0}^{T}B_{i}\log_{2}\left(1+\gamma_{i,u}^{\left(t\right)}\right)dt\;,\;for\;WiFi\; \end{array}\right\}$$

where ∝ denotes the α-type network and *T* is the overhead for the linked AP. It should be noted that utilizing (2) rather than (1) for network capacity estimates for the suggested approach (MCDM) may produce different results because the former focuses on an AP’s capacity inside the chosen network while the latter seeks to determine the capacity of the entire network (LiFi or WiFi).

Because the entire capacity of a network is divided among its APs, the number of users and their data rate requirements, and data consumption, a choice based only on an AP’s capacity may be affected by the number of APs used and the number of users connected. When different numbers of APs are employed in the hybrid system, which is typically the case, taking into account a single AP’s capacity results in the inequity of the network capacities between the LiFi and WiFi. Therefore, in this suggested MCDM technique, the network’s overall capacity is taken into account for the referred criterion in order to prevent this problem and make this study suitable for any number of Aps. For this hybrid LiFi system, which is challenged by the mobility element, the evaluation of the LiFi CDT value is crucial and significant. Different vertical HO issues were addressed using the two fundamental VHO systems, immediate vertical handover (I-VHO) and dwell vertical handover (D-VHO). A crucial statistic for the HO process, regardless of whether it is brought on by user mobility or channel obstructions, is the CDT, which is defined as the amount of time a user remains connected to an AP without being disconnected. In conclusion, the HHO/VHO selection requires using decision-making or optimization techniques to jointly take the channel quality, resource availability, and CDT into account [95]. The choice to switch over might be taken in the best interests of either the network as a whole or a specific user. The CDT was seen as one of the values that needed to be taken into account when creating the HO strategy. Additionally, the CDT was used to calculate the HO cost [96].

In addition, [15] describes and incorporates light-path blockage into the formulation of the CDT-based LB problem. The CDT value has an impact on the HO process, and a short CDT increases the difficulty of the HO process and the ping-pong effects [57]. We may evaluate the percentage of the time spent on an HHO in hybrid LiFi while taking mobility and obstructions into account by using the CDT [21].

A user’s CDT could change over time. This information may therefore be regularly updated and quantitatively measured. As a result, using the most recent CDT, the suggested procedure in [21] was regularly used. Keep in mind that a dynamic environment with fast-moving users causes the CDT value to be low. Calculating the percentage of time spent on the HO process can be done using the computation of the CDT that can be statistically gathered [96]. The speed of the user in hybrid LiFi networks varies. The study by [76] considered using 0.2, 0.5, and 1.4 m/s for user velocity. Another study by [97] used 1, 1.4, and 2 m/s for user speed, as the movement was based on the orientation-based random waypoint (ORWP) mobility model. However, some studies considered using a specific value of the user’s speed (such as 1, 2, or 5 m/s), while others considered using a range of movement where the speed/velocity is a random variable uniformly distributed between 0 and a maximum speed [18,96]. Moreover, we have summarized the user speed and/or user velocity used in previous studies in Table 2.

The D-VHO theory interacts with the CDT definition. In [99], a VHO decision-making algorithm based on FL was proposed. It was recommended to use this method for various optical wireless hybrid systems. It demonstrates the significance of I-VHO and D-VHO in achieving exemplary HO decisions with regard to packet transfer delay. The D-VHO had either a “short” or “long” waiting period.

**Figure 6:**
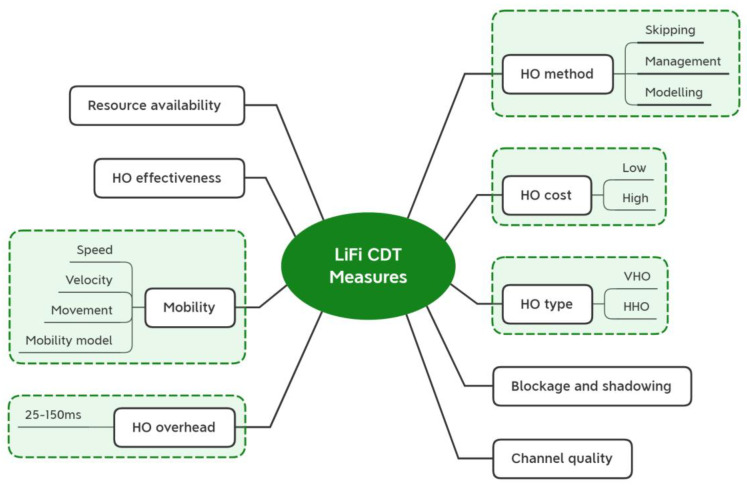
Factors involved in measurements of LiFi CDT.

The VHO decision procedure will be activated if an interruption happens in order to choose a dwell time prior to the VHO execution. The MT will restart its stopped transmission using the LOS optical channel if the LOS optical channel resumes before the dwell timer expires; otherwise, a VHO will be carried out. The elements that go into measuring the CDT are outlined in Figure 6. In light of the aforementioned facts, it follows that user mobility and obstructions have an impact on the remaining criteria, the occupation and occurrence rates, and *O_ccp_* and *O_ccr_*. Light-path bottlenecks are described by both criteria.

The degree of channel obstruction reflects how much the channel quality is impacted by the blockage. It was considered that, once a blockage occurred, the blockage degree had reached its maximum and that no method could provide any throughput in this situation.

The *O_ccp_* is specifically the percentage of time that a consumer experiences channel blockage. The user should remain in the LiFi network unless the *O_ccp_* of channel blockage is high, in which case they should always be connected to WiFi [15,100]. This is done for two reasons: first, it results in sporadic HOs; second, if such users continued to use LiFi, they would frequently go without service. Each user has an *O_ccp_* that is evenly divided between 0 and 1 [15]. However, the study by [100] indicated values for the *O_ccp_* of 0.2 and 0.8. When the *O_ccp_* was equal to 0.1, it was believed that light-path blockages happen once every minute. With that said, it is clear that the *O_ccp_* notion differs from the CDT in that the former refers to a user who is continuously blocked without a connection and the latter refers to a user who is continuously connected without a blockage.

The number of channel blockages that occur per unit of time is indicated by the variable *O_ccr_*. A user with a high *O_ccr_* of channel obstruction would experience frequent HOs if they switched to WiFi.

All users’ *O_ccr_* is gamma distributed with a 1 shape factor. The channel blockage occurrences for each user with a certain *O_ccr_* are presumed to follow a Poisson point process (SSS), which is typically used to mimic random events such as packet arrival at switches [101]. In the study by [15], *O_ccr_* was set to 10 times per minute (abbreviated as 10/min) while assessing system throughput. The results of their investigation showed that, as *O_ccr_* rises, the obtained throughput falls. The maximum throughput is achieved when *O_ccr_* = 0. When channel obstruction arises, it is worthwhile to switch users who have a low occurrence rate but a high occupation rate to WiFi. Note that the values of *O_ccp_* and *O_ccr_* will reach zero if no user has ever experienced a channel blockage. Two criteria result in a decline in throughput performance. All the aforementioned criteria values are summarized in Table 3; these values will be used throughout the rest of the study.

Some values in Table 3 are subject to logic and reasonability, while others come from previous knowledge. Specifically, LiFi CDT assumes each 1 **s** consists of 2 interval states, which refers to every time the CU makes a decision, refresh, and update, where the user might be experiencing an HO in the following state after the first. Therefore, the lowest value for the LiFi CDT could be 0.5 s, which represents 1 interval state, and the maximum is at 10 intervals (5 s). The WiFi capacity (protocol 802.11 n) is set to 120 Mbps using a 2.4 GHz carrier frequency and 20 MHz of bandwidth [100]. According to [15], the LiFi capacity can be set to a maximum of 447 Mbps for 10 users, 9 LiFi APs, with a maximum user speed of 5 m/s, and the minimum is supposed to be at least 427 Mbps.

The minimum value for the occurrence rate is set to 0, where some users may not experience any blockage per minute, and the occupation rate is set from 0 to 1 [15]. Since we have five criteria for this study, each criterion consists of five different ranges of values, it is important to consider all the possibilities that could occur in a dynamic environment where all values are impacted and susceptible to changes due to blockages and user mobility. Therefore, we have created all the possible combinations of all 5 items together with no duplicates in order to create groups that reflect every single combination of all criteria. A set of combinations denoted as Ɽ has been calculated as Ɽ = X^Y^, where X represents the number of criteria and Y represents the number of values of ranges for each criterion.

Thus, Ɽ = 5^5^ results in having 3125 sets of possible combinations. Each combination is considered as a rule (rule 1, rule 2, rule 3, …, rule 3125). For the full set of combinations, see Supplementary Materials. Since this research focuses on finding a solution for the APA that is caused by blockages and user mobility, a set of categories that consist of different values of mobility with different blockage degree estimations is designed and introduced, as shown in Table 4.

In order to simulate new-world examples, three categories are introduced where low, medium, and high blockage are considered, respectively. Each category is combined with different mobility values including low, medium, and high. Here, the values of each criterion given in Table 4 are set based on analysis of previous works of LiFi studies including algorithms, schemes, and mathematics. Each combination in Table 4 belongs to a rule from Ɽ.

Furthermore, the values of each criterion are estimated and set based on the ranges from Table 3. Moreover, each category in Table 4 belongs to a specific rule ID from Ɽ; therefore, it is identified accordingly, as shown in the column “Rule ID”. The estimation of the criteria value was evaluated and set based on the appropriate value corresponding to the values of user mobility and degree of blockage (DoB) from/in each category.

#### 3.1.5. Identifying the Decision-Making Alternatives

**Figure 7:**
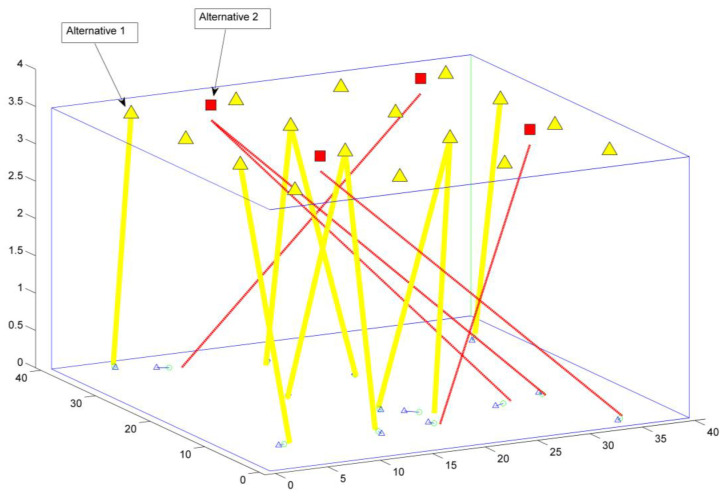
Example of DM alternatives in hybrid LiFi network.

In this stage, we can see the several options from which we can choose. System solutions consisting of 4 WiFi APs and 16 LiFi APs are depicted in Figure 7. WiFi APs are strategically positioned to cover the entire space. Each LiFi AP is a WiFi hotspot built into an LED lamp in a specific area. With the optical spectrum being reused by the LiFi APs, cell-to-cell interference is kept to a minimum. LiFi APs and WiFi APs here stand in for options 1, 2, …, etc., respectively. The data should be written in the alternative as Matrix A = [*Ai*], where *i* = 1 *…n*, which represents the number of alternatives [32].

To reach a determination, the AHP can generate the following comparison matrix: they perform a pairwise comparison of the criteria to establish their relative importance. In this stage, the importance of the criteria will be calculated in terms of their relative weight. C = [c_ij_] and the weight they carry in the decision-making process.

### 3.2. Weighting Phase

In this step, if we want to assign relative importance to each criterion, we need to create a pairwise comparison matrix, which is a matrix based on the decision-makers’ own subjective ratings of the possible pairings.

First, through verbal judgements such as “equally important,” “slightly more important,” “totally more important,” etc. and then through assigning values on a scale between 1 and 9, as shown in Table 5, which shows the importance degree of one criterion towards another criterion). To conclude, the AHP can generate the following comparison matrix:

(3) $$A=\left(\begin{array}{c} \begin{array}{ccc} x_{11} & x_{11} & \ldots \\ x_{21} & x_{22} & \ldots \\ : & : & \ldots \end{array}\\ \begin{array}{ccc} x_{n1} & x_{n2} & \ldots \end{array} \end{array}\;\begin{array}{c} \begin{array}{cc} \ldots & x_{11}\\ \ldots & x_{2n}\\ \ldots & : \end{array}\\ \begin{array}{cc} \ldots & x_{\mathrm{nn}} \end{array} \end{array}\right)\;\mathrm{where}\;,\left\{\begin{array}{c} x_{\mathrm{ii}}=1\\ x_{\mathrm{ji}}=\frac{1}{x_{\mathrm{ij}}} \end{array}\right.\;.$$

When comparing two criteria backwards, the significance value is the same as when comparing them head-on [32]. Next, we enter our information into a two-dimensional matrix, with n representing the number of decision criteria for which the table should contain the value that comes from comparing two criteria.

The ratios from 1/2 to 1/9 will be stored in a new pairwise comparison matrix, with the sum of each column representing intensities x, with x = 1 to 9 (integer) transformed into c via the following relations. For this, we used a linear scale, where c = x [102,103].

Evaluation of options by applying criteria to them and assigning weights accordingly. Similar to the technique for evaluating criteria, the outcomes of this comparison are stored in a square matrix with “N” components, where “N” is the number of alternatives under consideration. It has been shown in [32] that the number of matrices is always equal to the number of criteria. A typical pairwise comparison matrix is displayed in Table 6.

**Figure 8:**
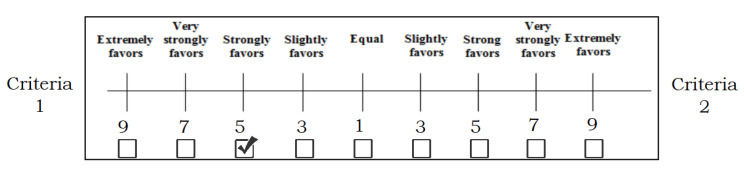
Sample of the questionnaire form.

A questionnaire based on pairwise comparisons was developed and sent out to an experts’ convenience sample that represented a variety of geographic locations. It was requested of the experts that they demonstrate their evaluations as well as the relative relevance of each criterion by using the nine scales as a comparison tool. In the assessment form that was given to several experts, Figure 8 provides an example of the criteria that can be used to make pairwise comparisons.

If n criteria are being utilized for evaluation, then the number of pairwise comparisons needed is *n* × (*n* − 1)/2. At this point, the AHP takes the pairwise comparison based on user preferences and judgements from the decision-making team and calculates the weight of importance of each criterion. In this study, the opinions and preferences of eight professionals with more than ten years of experience were gathered.

#### 3.2.1. Normalization for DM

Adjusting the relative merits of the alternatives is based on each criterion for making a choice. The ninth step presupposes that the alternatives’ relative rankings on each criterion will be converted into weights. Normalized values are calculated by dividing the comparison value by the total for the corresponding column [32]. Here, we will normalize

(4) $$a_{ij}=\frac{x_{ij}}{\sum_{i=1}^{n}x_{ij}}$$

with the normalized value **“**$a_{ij}$**”** being the result of a division by the sum of all the numbers in each column. Afterwards, the weights are computed by averaging the normalized values across rows and then used for the pairwise comparison between criteria; this involves converting the pairwise criterion into weights, where the normalized value was used, and the following condition holds true for the importance coefficients (the weight of decision criteria), which is done using Equation (5).

(5) $$A_{norm\;}\left(\begin{array}{c} \begin{array}{ccc} a_{11} & a_{11} & \ldots \\ a_{21} & a_{22} & \ldots \\ : & : & \ldots \end{array}\\ \begin{array}{ccc} a_{n1} & a_{n2} & \ldots \end{array} \end{array}\;\begin{array}{c} \begin{array}{cc} \ldots & a_{1n}\\ \ldots & a_{2n}\\ \ldots & : \end{array}\\ \begin{array}{cc} \ldots & a_{nn} \end{array} \end{array}\right)$$

#### 3.2.2. Calculation of All Priority Values (Eigenvector)

In order to assign relative importance to each criterion, the AHP pairwise comparison employs a series of mathematical calculations. After receiving the results from the pairwise comparisons, a reciprocal matrix is generated. The following formula can be used to get the weights of choice factor i:

(6) $$w_{i}=\frac{\sum_{j=1}^{n}a_{ij}}{n}\;\mathrm{and}\;\sum_{j=1}^{n}w_{i}=1,$$

where *n* is the total number of objects being compared. The AHP evaluation should be structured so that the weights are obtained according to the evaluator’s personal preferences.

#### 3.2.3. Calculation of CR

This is the step when we check for uniformity. Stepping through the steps [32] below will allow us to determine the consistency factor of the selection criterion matrix. According to the AHP definition of consistency, which is defined as “cardinal transitivity between judgements” [104], there should be no inconsistencies in the system. When using Equation (7) as a foundation, the first step is to determine the vector priority λmax, which is the product of the matrices of relative weight decision criteria and average weight decision criteria, where (c. k) represents the elements of the matrix–vector, which are the product of the “c” matrix and the “k” vector [32]. The following are the steps involved in the AHP method:

(7) $$\lambda max=\sum_{j=1}^{N}\frac{\left(c.j\right)}{N.kj}$$

The next step is to calculate a standard deviation of the stochastic uniformity coefficient. The rank of the analyzed matrix, denoted by the letter “*N*”, determines the average stochastic uniformity coefficient, denoted by the letter “*R*”:

(8) $$CI=\frac{\lambda max-N}{N-1}$$

Finding the uniformity coefficient is the third step. When using Formula (8), we get the following results for the **“***CI***”** uniformity coefficient: A fourth step is to calculate the matrices’ consistency factors. If the consistency relation (*CR*) is less than 0.10, then the matrix is consistent, and the weight vector may be computed with high confidence [29].

(9) $$CR=\frac{CI}{RI}$$

In this study, we will use the Alonson/Lamata linear fit, resulting in [74] the CR:

(10) $$CR=\frac{\lambda_{max}-N}{2.7699N-4.3512-N}$$

#### 3.2.4. Calculation of Row Geometric Mean Method (RGMM) and Weighted Geometric Mean Method (WGMM)

One of the most common techniques in AHP and MCDM studies [104] was used to assign relative importance to each criterion. Multiples of the weights of criteria and subcriteria (if needed) at the same hierarchical level were applied to the priority weights of criteria. Priorities $p_{i}$ are calculated using the row geometric mean method (RGMM). With the pairwise *N* × *N* comparison matrix A = $a_{ij}$, we calculate:

(11) $$r_{i}=exp\left[\frac{1}{N}\;\sum_{j=1}^{N}1n\left(a_{ij}\right)\right]={\left(\prod_{i=1}^{N}a_{ij}\right)}^{1/N}\;$$

and normalize:

(12) $$p_{i}=r_{i./}\sum_{i=1}^{N}r_{i}$$

#### 3.2.5. AHP Consensus Indicator (AHP-S*)

The aim of the AHP group consensus indicator proposed in [73] is to provide a numerical measure of the group’s ability to reach a unified opinion on an issue, or more specifically, to provide an estimate of the degree to which group members will prioritize different outcomes. This metric can take on values between 0 and 100. In this context, 0% represents no consensus at all and 100% represents total agreement. This metric is based on Shannon entropy [105], specifically the alpha and beta entropies, which are used to describe diversity [106]. It is a way to assess how much participants in a group share similar priorities, or how much their priorities overlap with one another. When all inputs are run through the RGMM, the combined Shannon alpha and beta entropies are used to determine the AHP consensus [73]. The indicator for consensus can be anywhere from 0% (total disagreement among decision-makers) to 100% (complete consensus between decision makers). *N* criteria, *K* participants/decision-makers. Interpretation of the AHP consensus indicator AHP-S*

(13) $$\mathrm{AHP}-S*=\left[M-exp\left(H_{\alpha min}\right)/exp(H_{\alpha max}\right]/\left[M-exp\left(H_{\alpha min}\right)/exp\left(H_{\lambda max}\right)\right]$$

$\mathrm{where}\;M=1/\exp\left(H_{\beta }\right)$ and $H_{\alpha ,\beta ,\gamma }$ is the α,β,γ Shannon entropy for the priorities of all K decision-maker participants. Then, we calculated the Shannon alpha entropy by the following formula:

(14) $$H_{\alpha }=\frac{1}{K}\sum_{j=1}^{k}\sum_{j=1}^{k}-p_{ij}1n\;p_{ij}$$

The Shannon gamma entropy is calculated as:

(15) $$H_{\alpha \gamma }=\sum_{j=1}^{k}-{\overset{´}{p}}_{j\;}1n\;{\overset{´}{p}}_{j\;}\mathrm{where}\;{\overset{´}{p}}_{j\;}=\frac{1}{N}\sum_{i=1}^{N}p_{ij}$$

Then, the Shannon beta entropy is calculated as:

(16) $$H_{\beta =}H_{\alpha \gamma }-H_{\alpha }$$

Aggregation is performed here by using the weighted geometric mean method (WGMM) [105], as follows:

(17) $$Z_{i}=\prod_{k=1}^{m}Z_{ik}^{w_{k}},i=1,2,\ldots ,n$$

### 3.3. Ranking Phase

In this section, the created DM is proposed including the alternatives for the APA process and their preferred AP. All criterion values have also been determined as a direct consequence of the comparison between Table 3 and Table 4. Based on the union of the criteria value estimates and the criteria value estimates used to create the decision matrix, the DM for the hybrid LiFi/WiFi network with APA options may be derived. In Table 7, the range values for each criterion are replaced by a single, fixed value calculated using the median of the corresponding range values in Table 3. A ranking of options based on values of criteria in various scenarios will be provided and debated, with reference to the decision matrix, categories, and rules (from Table 4) described previously. The criterion used to determine the placement will be categorized as either benefit criteria (BC) or cost criteria (CC), in which case the benefit criterion is thought to be the best when it is increased and the worst when it is dropped. When costs are minimized, however, they are deemed to be optimal, and the opposite is true when costs are increased. Table 8 shows the best and worst values for each case. Designing and modeling systems using the MCDM approach is aided by familiarity with the categorization of each criterion and the projected impact of each on the APA procedure. This idea of categorizing criteria was overlooked in earlier research on LiFi.

Since the nature of the variables and elements in the system is fixed, for instance, a BC cannot be changed to a CC, and vice versa, the researcher’s knowledge of the system is crucial to the determination and identification of each criterion as a BC or CC. Bear in mind that figuring out how to properly establish each criterion is crucial, for the simple reason that a mistake in identifying the criterion in this case could lead to false-positive ranking values for some or all criteria.

### 3.4. VIKOR Steps

**Figure 9:**
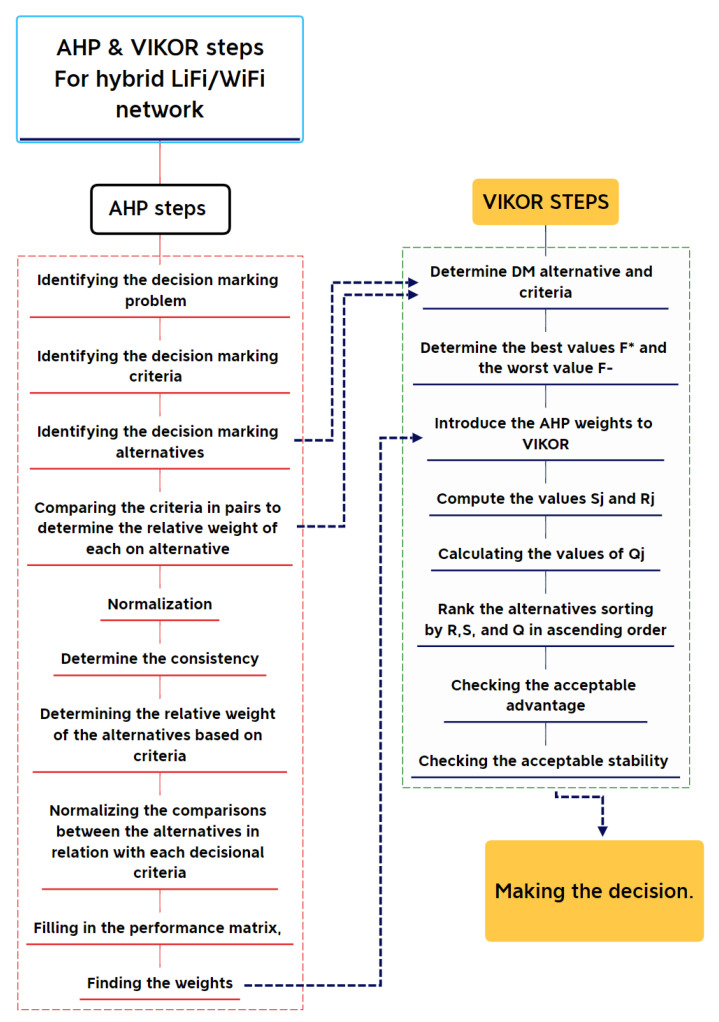
AHP–VIKOR integration steps.

**Figure 10:**
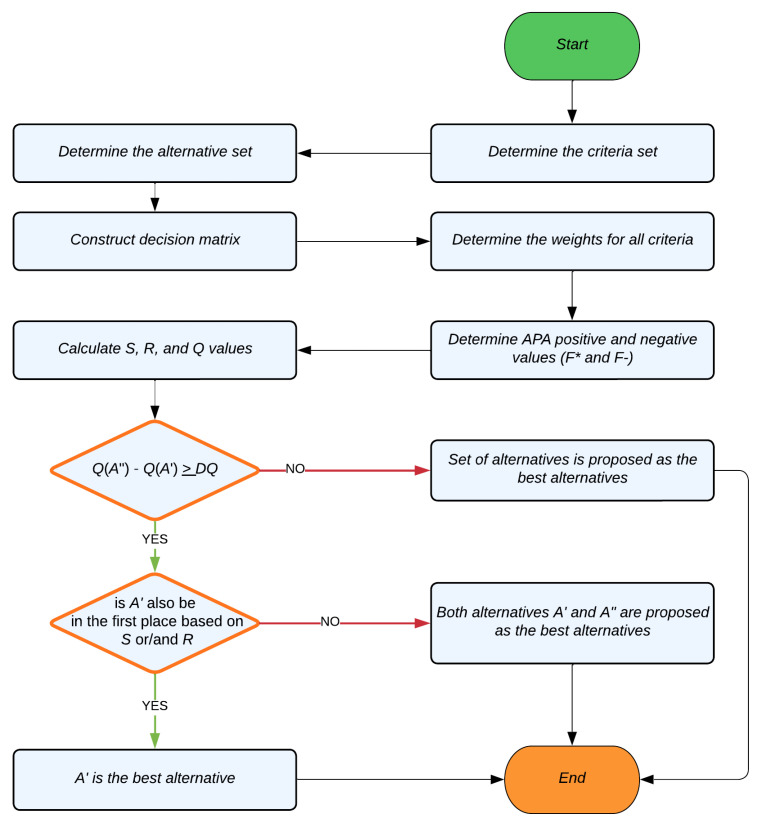
Flowchart of AHP–VIKOR methodology for evaluating satisfaction level.

To address VIKOR’s primary shortcoming—its inability to provide relative weights to evaluation criteria—the AHP method’s weighted criteria is employed here to determine the order of APs in the presented hybrid LiFi/WiFi network. All the possible alternative scores (APs) should be sorted from highest to lowest. Figure 9 illustrates how the AHP and VIKOR procedures work together. Before giving application steps, so to make it more easily understandable, the flowchart of the AHP–VIKOR method is presented in Figure 10. The VIKOR steps are explained as follows:

**Step 1**: Determining the DM used for the APA problem in this research. This process involves identifying *m* alternatives and *n* criteria and their corresponding values. The outcome is the DM intersecting all the criteria with their values across all the alternatives. The DM is presented in Table 7.

**Step 2**: Determination of the best values F* and the worst value *F-* for all criteria (*I =* 1,2,3,4…*n*). If the *i* value is represented as a benefit criteria, then we have to obtain the following formula to find F*:

(18) $$\int_{i}^{*}=max_{j\;}\int ij,\;$$

Otherwise, if the *i* value is represented as the cost criteria, then we have to obtain the following formula to find *F-*

(19) $$\int_{i}^{-}=min_{j\;}\int ij,\;$$

**Step 3**: In this process, the weights for each criterion (AHP weights) are introduced to VIKOR. A set of weights *w = w*_1_; *w*_2_; *w*_3_; …; *w_j_*; …; *w_n_*, from the decision-maker (expert) is accommodated in the DM; this set should be equal to 1. The resulting matrix can also be calculated as follows:

(20) $$WM=w_{i}^{\;}\left(f_{i}^{*}-f_{ij}^{*}\right)/f_{i}^{*}-f_{i}^{-})$$

This process will produce a weighted matrix as follows:

$$\left(\begin{array}{ccccccc} \left.w_{1}\left(f_{1}^{*}-f_{11}^{*}\right)/f_{1}^{*}-f_{1}^{-}\right) & \cdots & \left.w_{2}\left(f_{2}^{*}-f_{12}^{*}\right)/f_{2}^{*}-f_{2}^{-}\right) & & \cdots & \cdots & \left.w_{i}\left(f_{i}^{*}-f_{ij}^{*}\right)/f_{i}^{*}-f_{i}^{-}\right)\\ \vdots & \vdots & \vdots & \cdots & \cdots & \cdots & \vdots \\ \left.w_{1}\left(f_{1}^{*}-f_{21}^{*}\right)/f_{1}^{*}-f_{1}^{-}\right) & \cdots & \left.w_{2}\left(f_{2}^{*}-f_{22}^{*}\right)/f_{2}^{*}-f_{2}^{-}\right) & & \cdots & \cdots & \left.w_{i}\left(f_{i}^{*}-f_{ij}^{*}\right)/f_{i}^{*}-f_{i}^{-}\right)\\ & \vdots & & \ddots & & & \vdots \\ \left.w_{1}\left(f_{1}^{*}-f_{31}^{*}\right)/f_{1}^{*}-f_{1}^{-}\right) & \cdots & \left.w_{2}\left(f_{2}^{*}-f_{32}^{*}\right)/f_{2}^{*}-f_{2}^{-}\right) & & \cdots & \cdots & \left.w_{i}\left(f_{i}^{*}-f_{ij}^{*}\right)/f_{i}^{*}-f_{i}^{-}\right)\\ \vdots & \vdots & \vdots & \cdots & \cdots & \cdots & \vdots \\ \left.w_{1}\left(f_{1}^{*}-f_{41}^{*}\right)/f_{1}^{*}-f_{1}^{-}\right) & \cdots & \left.w_{2}\left(f_{2}^{*}-f_{42}^{*}\right)/f_{2}^{*}-f_{2}^{-}\right) & & \cdots & \cdots & \left.w_{i}\left(f_{i}^{*}-f_{ij}^{*}\right)/f_{i}^{*}-f_{i}^{-}\right) \end{array}\right)$$

**Step 4**: Compute the values *S_j_* and *R_j_* by the relations, where *wi* are the weights of criteria expressing their relative importance as follows:

(21) $$S_{j}=\sum_{j=1}^{n}W_{i}*\left(f_{i}^{*}-f_{ij}^{*})/f_{i}^{*}-f_{i}^{-}\right)$$

(22) $$R_{j}=MAX\;W_{i}*\frac{f_{i}^{*}-f_{ij}^{*}}{f_{i}^{*}-f_{i}^{-}}$$

**Step 5**: Calculating the values of *Q_j_*, *J* = (1,2,3,4 …; *J*)**,** where we will compute it by using the following relation:

(23) $$Q_{j}=\frac{v\left(S_{j}-S^{*}\right)}{S^{-}-S^{*}}+\frac{\left(1-v\right)\left(R_{j}-R^{*}\right)}{R^{-}-R^{*}}\;$$

where S^*^ Min *S_j_, S^−^* Man S_j_, and R^*^ Min R_j_, R^*^ Man *R_j_*, where the value *v* is the majority of criteria ”or\the maximum group utility”; in this research, *v* is equal to 0.5.

**Step 6**: Ranking the set of alternatives (*access points A1 to A11*) by sorting the values, *S*, *R*, and *Q* in ascending order, resulting in three different ranking lists.

**Step 7**: Proposing a compromise solution where the alternative (*A1*) is the best-ranked in accordance with the least Q value. This applies should both conditions be met, including where “*Acceptable Advantage*” and “*Acceptable Stability*” are satisfied. Where *A*′ is the second-ranked alternative in the list according to the *Q* value, *A*′ is the first-ranked alternative in the same list. Finally, for *DQ* = 1/(*m* − 1), *m* is the number of alternatives.

- C1. “Acceptable Advantage”;
- *Q* ($A^{\prime \prime }$) **−** Q(A**′**) ≥ DQ;
- C2. “Acceptable Stability”.

Alternative *A*′ must also be the best-ranked according to *S* or/and *R*. This compromise solution is stable in the decision-making process, which can use “*voting by majority rule*” (when *v > 0.5* is needed), “by consensus” (*v ≈ 0.5*), or “with veto” (*v < 0.5*). In case any of these two previous conditions are not satisfied, a set of compromise solutions are proposed, including the following

- Alternative *A*′ and *A*″ when only C2 is not satisfied, or
- Alternative *A*′ and *A*″ … *A^m^* if C1 is not satisfied and *A^m^* is determined using the relation *Q (*$A^{\left(m\right)}$*) − Q(A′)* < *DQ.*

## 4. Results

This section discusses the results of the prioritization for the proposed framework. Results from the DM are shown and discussed first, and then the results from the AHP method’s evaluation of the subjective weights of the criteria are presented. The multicriteria weights are then used in the VIKOR setups to determine the final ranking for the APs. Below is an illustration of the logical progression of the outcomes.

### 4.1. DM Results

In this section, to understand the impact of each criterion for the APA process in a hybrid LiFi/WiFi network, each criterion is taken individually as the base value for ranking the best and worst cases. This is because each criterion has a different influence on the system; therefore, a variety of different decisions can be made. In a conclusion, as shown in the column (rank) in Table 9, rank number 1 represents the best case while rank number 11 represents the worst case. Moreover, Table 9, Table 10, Table 11, Table 12 and Table 13 present the alternatives and their ranking for the different situations. Specifically, each of the mentioned tables uses a different criterion to provide a different ranking. This is to show the impact of using one criterion for making a decision compared to others. For example, Table 9 provides a ranking based on the usability of the first criteria (LiFi capacity), whereas alternative A4 has the best value based on the highest LiFi capacity, which is shown as the first rank.

On the other hand, alternative A7 has the worst value because it has obtained the lowest LiFi capacity and is considered the worst rank at level 11. This concept is reflected in Table 10 and Table 11 because they are classified as BC as well.

In Table 10, alternative A9 is considered the best at the first rank and A7 is the worst, and the same applies to Table 11. On the other hand, in Table 12 and Table 13, which are considered the CC, the lowest values represent the best alternative and are ranked as the best, while the highest values represent the worst alternatives and are ranked as the worst.

As a summary, Table 14 summarizes all the alternatives and their ranking based on the values presented in Table 9, Table 10, Table 11, Table 12 and Table 13 for all criteria. The ranking results in this section motivate the prioritization using AHP–VIKOR for the complete MCDM procedure. Based on the foregoing research, the MCDM technique was developed to consider all criteria simultaneously to accomplish the goal of this study, which would be to develop the proposed technique for optimal decision-making. The following section provides the criteria for weighting measurement by using the AHP method. After that, the ultimate VIKOR standings are displayed.

### 4.2. Weighting Result Based on AHP

After completing all of the procedures outlined in Section 3.2, the AHP method’s findings are presented. In this section, we show and debate the outcomes of the experts’ criteria judgements (CJs). The CJs are also transformed into a pairwise comparison matrix (PCM), the RGMM is calculated, and finally, the consolidation matrix (CM), which is the weighted geometric mean of all participants, is calculated. Finally, the results of the weights are presented.

The weights reveal the relative value of each attribute as determined by the opinions of eight experts. All experts are specialized in LiFi, VLC, and optical communication with around 10 years of experience. Their qualifications are as follows: (i) high-ranked universities from the UK, US, and Canada, and (ii) 71% of them were professors, 14% were postdoctoral research associations in the LiFi research area, and 14% were professors and chairs of departments. Table 15 displays the aggregate weighted findings from all experts, displaying their preferences for individual criteria based on a comparison to the other criteria. The judgement results show the correspondence of the experts to the comparison questionnaire designed by the authors of this study. To understand the value of the CJs provided by the experts, all the data of all criteria must be converted to PCM form. In order to calculate the final weights of each criterion for all participants, we will use the PCM values shown in Table 16.

The values of the RGMM are displayed in Table 17. Both the aggregate of individual judgements (AIJs) and the aggregate of individual priorities (AIPs) are taken into account in the AHP. When the RGMM is used for periodization, this finding ensures that both aggregation methods yield group consistency at least as good as the lowest individual consistency (AIJs and AIPs). It was demonstrated in [104] that the simpler of the two aggregation procedures (often the AIPs) satisfies the consistency condition; hence, this method can be used in practice to obtain the group priorities.

The final priority of the options for the two aggregation procedures (AIJs and AIPs) is produced when the RGMM periodization procedure is used. Table 18 displays the values of the consolidation matrix. If the weighted geometric mean method (WGMM) is used for aggregation and the rank-based maximum likelihood method (RGMM) is used for prioritization, then the group error for the judgement is equal to the geometric mean of the weights assigned to the individuals’ errors in making the judgement. Table 19 lists the RGMM-obtained priority vectors and their corresponding GCIs for all matrices. If the various decision makers’ judgements are of an acceptable level of inconsistency, then the group’s geometric consistency index using the RGMM periodization process will be within an acceptable level as well.

Finally, the AHP weight results are introduced, which consist of the normalized principal Eigenvector which calculates each criteria weight in a percentage manner, where the summation of the weight of all criteria should be 100%. In addition, the weight of each criterion represents the final value that will be used for the prioritization of the alternative using the VIKOR, where the summation of the final weights of all criteria should be equal to 1.

We can note that the priority vectors and the individual matrices are of acceptable inconsistency (GCI < 0.35); as can be seen, the obtained value of the GCI in this study is 0.33. As long as it stays below 10%, a CR of 9.1% is considered acceptable. The consistency ratio (CR) is a recommended measure of individual inconsistency that is included in the standard AHP, as per the work of Saaty [32]. The α value equals 0.1 and the Lambda value is 5.410.

The value 1.2 × 10^−09^ is the EVM check. In practice, the earned value management functions well when the project expenses are closely monitored and managed. Using this method to evaluate progress toward the deadline, however, leads to significant discrepancies. The earned value has been modified in several ways to account for these gaps in accuracy while assessing the progress on a project’s timeline. This tracking is best done using earned value management. The concept of earned value is associated with the measurement of the work (scope) performed at the proposed cost, and when it is combined with the joint measurement at the baseline (scope, schedule, and cost), it is referred to as earned value management (EVM) [107].

The criteria “LiFi capacity” obtains the highest value of weight at 0.5928, followed by 0.1968 for the “LiFi CDT”. On the other hand, the worst value of weight was obtained by the criteria “Occurrence rate” and “Occupation rate” at 0.0552 and 0.0445, respectively.

When analyzing a project’s progress, the earned value management takes into account more than just the most important activities. Included are both crucial and noncritical activities, although the critical path will have the most impact on how long the project takes as a whole. There is an Eigenvalue of 5.40995468. The absolute value of the Eigenvalues allows for a natural ordering of the Eigenvalues from the largest to the lowest. The largest absolute Eigenvalue is referred to as the maximal Eigenvalue. Eigenvalues are roots of polynomials and could be complex numbers. Fortunately, this is impossible for pairwise comparison matrices, so we can limit our search to only real values. The AHP consensus indicator (AHP-S*) is 77.8%, which is considered a high value. It is calculated based on the value of the consistency ratio (CR). The CR is a measure of the consistency of pairwise comparisons.

The AHP consensus indicator is a useful tool for ensuring the reliability and validity of the AHP process. By ensuring that the pairwise comparisons are consistent, the AHP process can produce reliable and valid results that accurately reflect the decision-maker’s preferences and priorities. There are several benefits to finding the AHP consensus indicator. Overall, finding the AHP consensus indicator is an important step in ensuring the reliability and validity of the AHP process, and it can help to improve the transparency, accountability, and trustworthiness of the decision-making process.

1. It helps to ensure the reliability and validity of the AHP process: By ensuring that the pairwise comparisons are consistent, the AHP process can produce reliable and valid results.
2. It helps to identify and correct inconsistencies: If the AHP consensus indicator is greater than 1.0, it indicates that the pairwise comparisons are inconsistent.
3. By identifying and correcting these inconsistencies, the decision-maker can ensure that the AHP process is producing accurate and reliable results.
4. It helps to improve the transparency and accountability of the decision-making process: By demonstrating that the AHP process has been conducted consistently and reliably, the decision-maker can increase the transparency and accountability of the process.
5. It helps to build trust in the decision-making process: By demonstrating the reliability and validity of the AHP process, the decision-maker can build trust in the process and the results.

### 4.3. VIKOR Prioritization Results

The findings of the APA’s final phase are presented here. The VIKOR approach can be used to compare and contrast alternatives once the criteria weights have been established. To achieve this, it is common to practice determining the following values for each alternative: The S-value is the sum of the normalized criteria values for the alternative and shows its “suitability” as a whole. The R-value is the difference between the alternative’s highest and lowest normalized criterion values; it is a measure of how the alternative ranks to others. The Q-value quantifies the “quality” of the alternative and is determined by adding up the S-values of all less-desirable alternatives. Using these values, you can then rank the alternatives based on their overall suitability, relative rank, and quality. The alternative with the lowest S-value, R-value, and Q-value is typically considered the best option. Table 20 shows the values of F* and *F−* that were calculated based on Equations (18) and (19). The values of F* for the benefit criteria are 446.000, 84.000, and 4.800 Mbps for the LiFi capacity, WiFi capacity, and LiFi CDT, respectively. The values of *F*−are 0.100 and 0.100 s/min for the occurrence and occupation rates, respectively. The overall weights will be introduced to the VIKOR configurations from the AHP method, and the VIKOR results will be calculated for these values, in addition to the values of *S*, S*, *S−*, *R*, R*, *R−*, and *Q*, as shown in Equations (21)–(23) in Table 21.

APs are ranked from highest to lowest by the Q score. Prioritization findings are introduced generally. Table 22 lists the final VIKOR prioritization results. It reveals that alternative A1 (access point) has the lowest value of *Q*, which means that it has best condition and the highest priority for the APA and is ranked first. According to the decision matrix in Table 7, A1 means the user will be assigned to the LiFi network, with the LiFi capacity of 444, the WiFi capacity of 41, 4.8 of LiFi CDT, the occurrence rate of 2, and 0.1 for the occupation rate.

The second-best AP is A9, wherein the user will be assigned to the LiFi. Users are also assigned to the LiFi for the following alternatives: A2, A10, and A4. That makes it a total of five alternatives assigned to the LiFi for the rank orders 1, 2, 3, 4, and 5. Furthermore, the remaining ranking orders, including 6, 7, 8, 9, 10, and 11, belong to A6, A3, A8, A11, A5, and A7, respectively, and set the users’ connection to the WiFi with a total of six APs.

Alternative A7 (access point) has the highest value of *Q*, which means that it has worst condition and the lowest priority for the APA and is ranked last.

According to the decision matrix in Table 7, A7 means the user will be assigned to the WiFi network.

Aside from having the rank by the Q values, it is also essential to determine the rank by S and R to satisfy in VIKOR the step 6 requirements. It is indicated that the majority of ranks are consistent, including the best rank (*A1*) and the worst rank (*A7*), with different *S*, *R*, and *Q* values in between, but all of them follow the VIKOR rules. On the other hand, slight ranks were different, including (*A8*) and (*A11*). Upon the completion of this step, the result of the last VIKOR step 7 is warranted.

According to this step, a compromise solution is proposed while meeting the “*Acceptable Advantage*”, and “*Acceptable Stability*” conditions mentioned above. When identifying the first condition, the results are (0.146 – 0.071) = 0.0752 ≥ 0.1. Based on these results, it is clearly seen that C1 was not met, and it is essential to check the C2. From the results, obtained, it is essential that, in addition to being the best according to Q value, *A’* must also be the best-ranked according to *S* or/and *R.* As seen in Table 22, *A1* was the best in terms of the *Q, S*, and *R* values, being the lowest, and therefore satisfying the C2 requirements. It is well-known in VIKOR that, if a condition is not met, a series of compromise solutions are presented, one for C1 and one for C2. In this research, only C1 was not met, and therefore its compromise solution is applied.

In such a case, the next value of *Q (*$A^{\left(3\right)}$*) − Q (*$A^{\left(1\right)}$*)* was applied to result in (0.180 − 0.071) = 0.1093 < 0.1. In such a scenario, it is clearly seen that the alternative *A9* (*second-ranked*) enters a set of compromise solutions because the first-ranked alternative *A1* from the ranking list does not have a “*Sufficient Advantage*” over the third-ranked alternative (*A2*). Other alternatives do not need to be tested according to the stated condition. Based on the obtained results, the final solution is defined by a set of compromise solutions in which there are alternatives *A1* and *A9*. In this case, the decision-maker can choose *A1−*APA (LiFi AP—RULE 270), and when switching to another AP, the most viable choice is *A9* (LiFi AP—RULE 813). It can be seen that the above results are different in comparison with the results presented in Table 9-14 due to the use of the VIKOR unique prioritization mechanism. In addition, these results were observed based on the weights given by the eight experts. It is significant to remember that the VIKOR technique does not offer a singular solution because the outcomes depend on the weights given to the criteria and how significant each one affects the final selection. To ensure that the results appropriately reflect the decision makers’ objectives, it is crucial to carefully analyze the criteria and weights utilized in the analysis.

In this context, the AHP weight results reveal that the LiFi capacity, LiFi CDT, and WiFi capacity have high values compared with the other criteria, as given in Table 19, which form 59.28%, followed by 19.68% and 11.07% of the total normalized main Eigenvector. These values set such criteria as the top priority and can alter the prioritization for the APA process. The VIKOR method’s prioritizing outcomes, however, demonstrate the most significance. As can be seen, the VIKOR gave APs with a lower Q value a higher priority. According to the VIKOR theory, the optimal solution will have the lowest possible Q value. The lowest values are found in the rates of occurrence (5.52%) and occupation (4.45%). Multiple tests showed that the recommended approach worked. Assigning users to the AP in hybrid LiFi networks is, therefore, possible using the proposed prioritization system. The difficulties can be dealt with by making use of integrated AHP–VIKOR.

In the above table, the difference of the ranking between the use of individual criteria without the MCDM and the integrated AHP–VIKOR method can be seen, where the best ranking with rule 270 is classified as G1 (from Table 4), which consists of low blockage and low user mobility. Rule 270 consists of high LiFi capacity and high LiFi CDT with a low occupation rate, which is represents A1 (LiFi). In addition, the second-best ranked AP holding the rule 813 is classified as G3, which has medium blockage and high user speed. It consists of high LiFi and WiFi capacities with medium LiFi CDT, occupation, and occurrence rates, which represents A9 (LiFi).

Finally, the worst-ranked AP with rule 3106 is classified as G2, which consists of high blockage and low user speed. It consists of low LiFi and WiFi capacities and low LiFi CDT, in addition to high occupation and occurrence rates, which represents A7 (WiFi). The first, second, third, fourth, and fifth ranking assign the user to the LiFi AP, while the rest of the rankings, from the sixth to the eleventh, are assigned to the WiFi AP.

At last, the evaluation and validation of the constructed weights from the viewpoint of wireless communication revealed that useful criteria can be offered, and the effects of these weights can be observed. Experts and researchers in the field may use the proposed technique as a guide if they decide to add new criteria or adjust the weights of the existing ones.

The best alternative that resulted in LiFi assignment refers to the fact that the assigned user had different values of user mobility and blockages than the worst alternative that assigns the user to WiFi. This is due to the fact that the findings obtained with the suggested method are distinct from those obtained with more conventional techniques. The findings of the APA before using the AHP–VIKOR based on individual criteria only are presented in Table 23, and these results are contrasted to the results obtained after using the AHP–VIKOR with the weighted criteria.

### 4.4. Sensitivity Analysis

Based on the aforementioned APA ranks and criteria weights discussed, it became clear that criteria weights significantly affect the assignments of the hybrid LiFi network amongst users, and therefore the final rank by the VIKOR; it is, therefore, essential to evaluate the VIKOR-based rank and the criteria weight changes and their effect on the process. Accordingly, an evaluation of the ranking outcomes of the developed integrated AHP–VIKOR approach is carried out in this section. There are several tools used in the literature to assess the robustness of the integrated MCDM work; amongst these is sensitivity analysis.

Many studies in the literature, such as [36], have used this assessment approach to measure the sensitivity of the criteria weights and analyze its change. Sensitivity analysis estimates the impact of the most significant criteria in terms of their weight on the results of the APA, and it requires different scenarios for the weight changes. There are different ways to do sensitivity analysis; in this study, we chose to base it on the ratio aspect, where benefit criteria equally get a specific percentage of the weight, while the cost criteria get the remaining percentage.

The following are the percentages and how we performed the sensitivity analysis, as seen in Table 24. As a proof of concept, sensitivity analysis [108] was carried out to measure the weight changes and their effect on the ranking over five scenarios, which result in the generation of new weight values, as shown in Table 25.

As seen from Table 24 and Table 25, we relied on ratio and percentages, where in scenario 1, the BC gets 90% of the weight distributed equally over its three criteria, wherein, when dividing 90% by three, the results should be equal for the three criteria as the LiFi capacity 0.9/3 = 0.3, as well for the WiFi capacity and the LiFi CDT. On the other hand, the CC gets 10% divided by the number of cost criteria, which is two, wherein 10% is divided by two, and the results will be 0.1/2 = 0.05 for the occurrence rate as well as the occupation rate.

**Figure 11:**
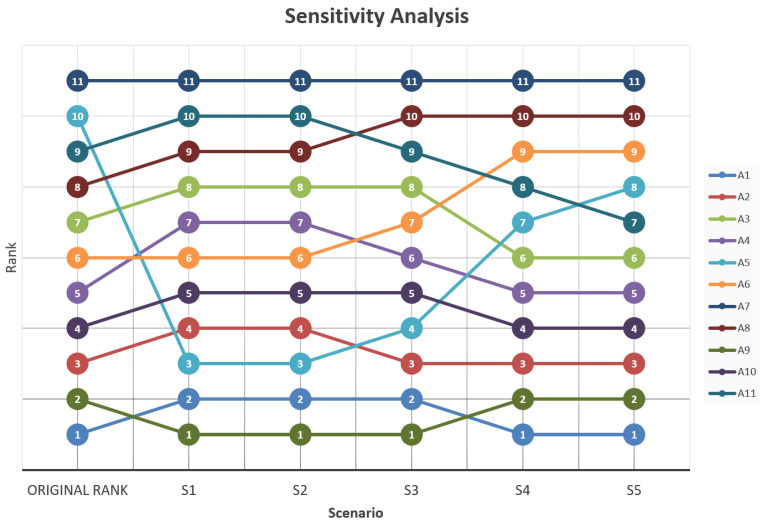
The new ranking of alternatives by sensitivity analysis.

It should be noted that, in each presumed scenario, the total percentage of the CC and BC should be 100%, which means the BC values are assumed to be equal 80%, 70%, 60%, and 50% for scenarios 2, 3, 4, and 5, respectively. The CC values are complementary to the BC towards the remaining 100%, which makes them 20%, 30%, 40%, and 50% for scenarios 2, 3, 4, and 5, respectively. Therefore, for scenario 2, the BC is assumed to be 80% and the CC is 20%. Here, all the BC criteria results are calculated as 0.8/3 = 0.266666667 and the CC are 0.2/2 = 0.1. In scenarios 3, 4, and 5, the BC values are as follows: 0.7/3, 0.6/3, and 0.5/3, which results in 0.233333333, 0.2, and 0.166666667, while the CC values are as follows: 0.3/2, 0.4/2, and 0.5/2, which results in 0.15, 0.2, and 0.25. Each scenario of the weights will be used to find the new priority of alternatives for the same set of criteria that are computed using the VIKOR again to find the new values for *S*, *R*, and *Q*, as shown in Table 26. The new rankings for the alternatives by the sensitivity analysis are shown in Table 27 and Figure 11.

Given that weight sensitivity changes in each category, further logic and discussion are required. It can be seen that the best “Original Rank” was *A1* followed by A9. In the first scenario (*S1*), the best ranking was attributed to *A9*, which means the user is connected to the LiFi AP, followed by the second-best ranking, which is *A1*, which is also a LiFi AP. The third-best ranking was *A5*, which assigns to the WiFi AP and implies a change in the AP compared with the original. On the other hand, the worst ranking obtained for S1 is *A7*, which is the WiFi AP and means no change compared to the original ranking.

For scenarios 1, 2, and 3, the first-best ranking was similar to the A9 alternative, which is the LiFi AP, followed by alternative A1 as the second-best ranking for the same three scenarios. The third-best ranking for S2 remains the same as S1, which is A5 (WiFi AP), while the third ranking for S3 shifted to A2, which is the same as the original, which is the LiFi AP. For a better illustration, the following Figure 11 is presented.

As seen from Figure 10, the new ranking result indicated some notable changes. It is clear that the best (first) ranking for S4 and S5 remains the same as the original, where *A1* assigns the user to the LiFi AP. The same concept applies on the second-best ranking, A9 (LiFi AP), for both scenarios: S4 and S5. The third-best rank, A2 (LiFi AP), was consistent for three scenarios, S4, S5, and S3, with the original rank. Finally, the worst ranking was associated with A7 for all scenarios and the original (WiFi AP).

Some AP ranking results were consistent with the original and some were different across the scenarios. The rank consistency is considered 100% for unchanged ranks when compared to the original, where in S4 and S5, the first five ranked alternatives (*A1*, *A9*, *A2*, *A10*, and *A4*) and the worst alternative (*A7*) ranking results remain the same as the original. It can be seen that the weight changes of the five evaluation criteria across the five new scenarios can notably affect the APA mechanism in a dynamic hybrid LiFi network. Such a case can shed light on the fact that weight importance could pose a big issue. The discussed scenarios of criteria changing prove that the importance of weights should be considered for the evaluation criteria because it can impact the final ranking results, and therefore plays a significant role for choosing the optimal AP for moving users with the existence of blockages and making the best possible decisions.

## 5. Conclusions

A LiFi/WiFi hybrid network in the indoor environment is considered in this study with multiple LiFi and WiFi APs. This hybrid network may provide superior performance to existing wireless options. However, with user mobility, shadowing, and blockages of the LOS, the system performance might be compromised. Therefore, HO and APA modeling is very crucial to optimize users’ QoS. A decision-making framework was designed and developed in this study for the first time for the APA and HO management in LiFi networks. The proposed method aims to assign the users to the best possible APs.

The AHP approach was used to subjectively weigh the importance of different criteria for such domains, with eight experts providing their assessments. The work provided is capable of distinguishing between situations with high and low user mobility and blockages. Following the comparable processes outlined in this research, the proposed framework can be adjusted to adopt, extend, or modify the criteria or subjective evaluation from LiFi specialists. Following the comparable processes outlined in this research, the proposed framework can be adjusted to adopt, extend, or modify the criteria or subjective evaluation from LiFi specialists. Sensitivity analysis was added to assess the stability of an optimal solution under changes in the weights. The approach presented in this work opens a new pathway towards multicriteria decision-making to solve different issues such as APA, HO, resource allocation, LB, etc.

The findings of this research will support dynamic LiFi networks with multiple users in terms of delivered data rates and stability. Despite the significance of this research contribution, there are some limitations which can be considered in future work. Firstly, the criteria aggregation was done using the arithmetic mean, which is common in MCDM research; however, other aggregation techniques can be considered and compared in future MCDM APA selection cases. Another limitation of this work is only considering APA selection challenges related to user mobility, speed, and blockages; in the future, other aspects of the APA selection can be considered separately or with those used in this research. The used integrated methodology in this research can be enhanced and applied to other types of hybrid LiFi networks.

## Figures and Tables

**Table 1:** Brief synopsis of related literature, including comparison to our own work.

Ref	Proposed Methods	Hybrid Network Types	Objective	Problem Considered				Issues Considered				Implementation			Other				
				LB	APA	HO		Blockage	Shadowing	Interferences	Mobility	Simulation only	Mathematics only	Simulation and Mathematics	Criteria evaluation	Weighted criteria	Prioritize criteria	DM	MCDM
						VHO	HHO												
[59]	A novel dynamic handover scheme based on fuzzy logic (FL)	RF–LiFi	Reduce the HO overhead	Yes	No	Yes	Yes	Yes	Yes	N/A	Yes	No	No	Yes	Yes	No	No	Yes	No
[60]	Two-stage APS based on FL	WiFi–LiFi	Reduce complexity and achieve higher throughput	No	Yes	No	No	No	No	N/A	No	No	No	Yes	Yes	No	No	Yes	No
[21]	A novel algorithm based on FL and a novel load-balancing scheme	WiFi–LiFi	Reduce the computational complexity and achieve a higher throughput	Yes	Yes	Yes	Yes	Yes	Yes	N/A	Yes	No	No	Yes	Yes	No	No	Yes	No
[63]	A two-phase APS scheme is proposed based on FL	mmWave–WiFi–LiFi	Reduce the computational complexity and achieve a higher throughput	Yes	Yes	No	No	Yes	No	Yes	No	No	No	Yes	Yes	No	No	Yes	No
[62]	A fuzzy logic (FL) and fuzzy rule-based artificial neural network (ANN) handover decision algorithms	WiFi–LiFi	To tackle the frequent handover experienced in the hybrid LiFi/WiFi network and reduce the complexity	No	Yes	Yes	No	No	No	No	Yes	No	No	Yes	Yes	No	No	Yes	No
[61]	Fuzzy logic scheme for APA	OCC–LiFi	APA+reduced complexity and enhanced QoS	No	Yes	Yes	No	No	No	Yes	No	No	No	Yes	Yes	No	No	Yes	No
Our study	AHP method using Shannon Entropy and VIKOR	WiFi–LiFi	APS, priority, weighted criteria, enhance QoS, reduce complexity, and boost system performance	Yes	Yes	Yes	Yes	Yes	Yes	N/A	Yes	Yes	Yes	Yes	Yes	Yes	Yes	Yes	Yes

**Table 2:** User speed/velocity values used in previous studies in LiFi networks.

Ref.	User Mobility		Network Topology
	Type	Value (m/s)	
[76]	User velocity	0.2, 0.5, and 1.4	Multitier LiFi Networks
[97]	User speed	1, 1.4, and 2	LiFi and mmWave networks
[21]	User speed	0–5	Hybrid LiFi and WiFi Networks
[18]	User speed	0–2.5	Hybrid LiFi and WiFi Networks
[96]	User velocity	0–2, and 0–5	Hybrid LiFi and WiFi Networks
[54]	User movement	0–1	Hybrid LiFi and WiFi Networks
[98]	Moving speed	0, 0.5, 1, 1.5, and 2	LiFi and WiFi
[79]	User speed	1	LiFi-based access networks
[55]	Node speed	0.3–0.7	Hybrid WiGig/LiFi Network

**Table 3:** Criteria values used for the decision matrix.

Criteria	Measurement Units	Values				
		Low	Not Low	Medium	Not High	High
LiFi capacity	Mbps	0–427	427–432	432–437	437–442	442–447
WiFi capacity	Mbps	0–10	10–30	30–60	60–90	90–120
LiFi CDT	Time (s)	0– 0.5	0.6–1.625	1.626–2.75	2.76–3.875	3.876–5
Occurrence rate	n/min	0–0.1	0.1–5	5–10	10–15	15–20
Occupation rate	n	0–0.1	0.1–0.25	0.25–0.5	0.5–0.75	0.75–1

**Table 4:** Categories, criteria value estimations, and rules assignment.

Categories		LiFi Capacity	WiFi Capacity	LiFi CDT	Occurrence Rate	Occupation Rate	Rule ID (Ɽ from Supplementary Materials)
**Category 1(G1)**	Low blockage; Med–High speed	High	Med	High	Not low	Low	Rule 270
		Med	Not high	Med	Low	Not low	Rule 1449
	Low blockage; Lower speed	High	Med	Low	Not low	Low	Rule 370
		High	Not low	Not low	Low	Not low	Rule 474
**Category 2(G2)**	High blockage; Med–High speed	Low	Med	Not high	Not high	High	Rule 2781
		Not low	Not low	Med	High	Not high	Rule 2302
	High blockage; Lower speed	Low	Low	Low	Not high	High	Rule 3106
		Not low	Not low	Not low	High	Not high	Rule 2327
**Category 3(G3)**	Med blockage; High speed	Not high	Not high	Med	Med	Med	Rule 813
	Med blockage; Med speed	Med	Med	Not low	Med	Med	Rule 1588
	Med blockage; Low speed	Not high	Med	Low	Med	Med	Rule 988

**Table 5:** Nine scales of pairwise comparisons.

Level of Importance	Description	Elaboration
1	Equal importance	Equal importance two different actions contribute equally to the goal.
3	Weak importance	Experience and judgement slightly favor one action over another.
5	Essential or strong importance	Expertise and judgment strongly favor one action over another.
7	Demonstrated importance	Action is highly preferred and its majority is seen in certainty.
9	Absolute importance	The evidence favoring one action over another is of the highest possible standard.
2, 4, 6, 8	Intermediate values between the two adjacent judgments	If a compromise is required.

**Table 6:** Pairwise comparison sample.

	LiFi Capacity	WiFi Capacity	LiFi CDT	Occurrence Rate	Occupation Rate
**LiFi capacity**	W_1_/W_1_	W_1_/W_2_	W_1_/W_3_	W_1_/W_4_	W_1_/W_5_
**WiFi capacity**	W_2_/W_1_	W_2_/W_2_	W_2_/W_3_	W_2_/W_4_	W_2_/W_5_
**LiFi CDT**	W_3_/W_1_	W_3_/W_2_	W_3_/W_3_	W_3_/W_4_	W_3_/W_5_
**Occurrence rate**	W_4_/W_1_	W_4_/W_2_	W_4_/W_3_	W_4_/W_4_	W_4_/W_5_
**Occupation rate**	W_5_/W_1_	W_5_/W_2_	W_5_/W_3_	W_5_/W_4_	W_5_/W_5_

**Table 7:** Decision matrix for hybrid LiFi with APA possibilities.

	Criteria	C1	C2	C3	C4	C5
Alternatives		LiFi Capacity	WiFi Capacity	LiFi CDT	Occurrence Rate	Occupation Rate
A1	LiFi	444	41	4.8	2	0.1
A2	LiFi	435	73	1.9	0.1	0.24
A3	WiFi	445	35	0.4	3	0.1
A4	LiFi	446	17	0.8	0.1	0.21
A5	WiFi	354	59	3.3	13	0.88
A6	WiFi	428	22	1.7	26	0.55
A7	WiFi	246	6	0.36	11	0.93
A8	WiFi	429	14	1.2	28	0.62
A9	LiFi	439	84	2.5	7	0.34
A10	LiFi	435	46	1.4	6	0.44
A11	WiFi	440	32	0.21	8	0.37

**Table 8:** Benefit and cost criteria classification.

Criteria	Benefits Criteria	Cost Criteria
LiFi capacity	✔	
WiFi capacity	✔	
LiFi CDT	✔	
Occupation rate		✔
Occurrence rate		✔

**Table 9:** Using Criteria #1 (LiFi capacity) for ranking the best and worst alternatives.

Scenario 1: LiFi Capacity							
Alternative	LiFi Capacity	WiFi Capacity	LiFi CDT	Occurrence Rate	Occupation Rate	Rule Based on Ɽ	Rank
A1	444	41	4.8	2	0.1	Rule 270	3
A2	435	73	1.9	0.1	0.24	Rule 1449	6
A3	445	35	0.4	3	0.1	Rule 370	2
A4	446	17	0.8	0.1	0.21	Rule 474	1
A5	354	59	3.3	13	0.88	Rule 2781	10
A6	428	22	1.7	26	0.55	Rule 2302	9
A7	246	6	0.36	11	0.93	Rule 3106	11
A8	429	14	1.2	28	0.62	Rule 2327	8
A9	439	84	2.5	7	0.34	Rule 813	5
A10	435	46	1.4	6	0.44	Rule 1588	7
A11	440	32	0.21	8	0.37	Rule 988	4

**Table 10:** Using Criteria #2 (WiFi capacity) for ranking the best and worst alternatives.

Scenario 2: WiFi Capacity							
Alternative	LiFi Capacity	WiFi Capacity	LiFi CDT	Occurrence Rate	Occupation Rate	Rule Based on Ɽ	Rank
A1	444	41	4.8	2	0.1	Rule 270	5
A2	435	73	1.9	0.1	0.24	Rule 1449	2
A3	445	35	0.4	3	0.1	Rule 370	6
A4	446	17	0.8	0.1	0.21	Rule 474	9
A5	354	59	3.3	13	0.88	Rule 2781	3
A6	428	22	1.7	26	0.55	Rule 2302	8
A7	246	6	0.36	11	0.93	Rule 3106	11
A8	429	14	1.2	28	0.62	Rule 2327	10
A9	439	84	2.5	7	0.34	Rule 813	1
A10	435	46	1.4	6	0.44	Rule 1588	4
A11	440	32	0.21	8	0.37	Rule 988	7

**Table 11:** Using Criteria #3 (LiFi CDT) for ranking the best and worst alternatives.

Scenario 3: LiFi CDT							
Alternative	LiFi Capacity	WiFi Capacity	LiFi CDT	Occurrence Rate	Occupation Rate	Rule Based on Ɽ	Rank
A1	444	41	4.8	2	0.1	Rule 270	1
A2	435	73	1.9	0.1	0.24	Rule 1449	4
A3	445	35	0.4	3	0.1	Rule 370	9
A4	446	17	0.8	0.1	0.21	Rule 474	8
A5	354	59	3.3	13	0.88	Rule 2781	2
A6	428	22	1.7	26	0.55	Rule 2302	5
A7	246	6	0.36	11	0.93	Rule 3106	10
A8	429	14	1.2	28	0.62	Rule 2327	7
A9	439	84	2.5	7	0.34	Rule 813	3
A10	435	46	1.4	6	0.44	Rule 1588	6
A11	440	32	0.21	8	0.37	Rule 988	11

**Table 12:** Using Criteria #4 (occurrence rate) for ranking the best and worst alternatives.

Scenario 4: Occurrence Rate							
Alternative	LiFi Capacity	WiFi Capacity	LiFi CDT	Occurrence Rate	Occupation Rate	Rule Based on Ɽ	Rank
A1	444	41	4.8	2	0.1	Rule 270	3
A2	435	73	1.9	0.1	0.24	Rule 1449	1
A3	445	35	0.4	3	0.1	Rule 370	4
A4	446	17	0.8	0.1	0.21	Rule 474	2
A5	354	59	3.3	13	0.88	Rule 2781	9
A6	428	22	1.7	26	0.55	Rule 2302	10
A7	246	6	0.36	11	0.93	Rule 3106	8
A8	429	14	1.2	28	0.62	Rule 2327	11
A9	439	84	2.5	7	0.34	Rule 813	6
A10	435	46	1.4	6	0.44	Rule 1588	5
A11	440	32	0.21	8	0.37	Rule 988	7

**Table 13:** Using Criteria #5 (occupation rate) for ranking the best and worst alternatives.

Scenario 5: Occupation Rate							
Alternative	LiFi Capacity	WiFi Capacity	LiFi CDT	Occurrence Rate	Occupation Rate	Rule Based on Ɽ	Rank
A1	444	41	4.8	2	0.1	Rule 270	1
A2	435	73	1.9	0.1	0.24	Rule 1449	4
A3	445	35	0.4	3	0.1	Rule 370	2
A4	446	17	0.8	0.1	0.21	Rule 474	3
A5	354	59	3.3	13	0.88	Rule 2781	10
A6	428	22	1.7	26	0.55	Rule 2302	8
A7	246	6	0.36	11	0.93	Rule 3106	11
A8	429	14	1.2	28	0.62	Rule 2327	9
A9	439	84	2.5	7	0.34	Rule 813	5
A10	435	46	1.4	6	0.44	Rule 1588	7
A11	440	32	0.21	8	0.37	Rule 988	6

**Table 14:** Ranking comparison of all criteria.

Alternative	Rank Based on 1st Criteria	Rank Based on 2nd Criteria	Rank Based on 3rd Criteria	Rank Based on 4th Criteria	Rank Based on 5th Criteria
A1	3	5	1	3	1
A2	6	2	4	1	4
A3	2	6	9	4	2
A4	1	9	8	2	3
A5	10	3	2	9	10
A6	9	8	5	10	8
A7	11	11	10	8	11
A8	8	10	7	11	9
A9	5	1	3	6	5
A10	7	4	6	5	7
A11	4	7	11	7	6

**Table 15:** CJ results obtained from the experts.

	Q1		Q2		Q3		Q4		Q5		Q6		Q7		Q8		Q9		Q10	
	LiFi capacity	WiFi capacity	LiFi capacity	LiFi CDT	LiFi capacity	Occurrence rate	LiFi capacity	Occupation rate	WiFi capacity	LiFi CDT	WiFi capacity	Occurrence rate	WiFi capacity	Occupation rate	LiFi CDT	Occurrence rate	LiFi CDT	Occupation rate	Occurrence rate	Occupation rate
EXPERT 1	-	5	-	9	7	-	1	1	9	-	5	-	5	-	9	-	7	-	7	-
EXPERT 2	9	-	7	-	7	-	7	-	-	5	5	-	5	-	5	-	7	-	1	1
EXPERT 3	7	-	9	-	5	-	7	-	-	7	5	-	5	-	9	-	7	-	1	1
EXPERT 4	7	-	7	-	7	-	9	-	-	7	-	5	-	5	-	7	9	-	1	1
EXPERT 5	9	-	9	-	7	-	9	-	-	7	7	-	5	-	5	-	9	-	1	1
EXPERT 6	9	-	7	-	9	-	7	-	-	7	7	-	5	-	3	-	9	-	1	1
EXPERT 7	9	-	5	-	7	-	9	-	-	5		-	9	-	7	-	5	-	1	1
EXPERT 8	7	-	5	-	7	-	7	-	-	5	3	-	5	-	9	-	5	-	1	1

**Table 16:** PCM for each expert of all criteria and the CM for all participants.

	8 = k Number of Participants 5 = n Number of Criteria										
EXPERT ID	LiFi Capacity	WiFi Capacity	LiFi CDT	Occurrence Rate	Occupation Rate	EXPERT ID	LiFi Capacity	WiFi Capacity	LiFi CDT	Occurrence Rate	Occupation Rate
**Expert 1**	**1**	1/5	9	7	1	**Expert 5**	**1**	9	9	7	9
	5	**1**	9	5	9		1/9	**1**	1/7	7	5
	1/9	1/9	**1**	7	9		1/9	7	**1**	5	9
	1/7	1/5	1/7	**1**	7		1/7	1/7	1/5	**1**	1
	1	1/9	1/9	1/7	**1**		1/9	1/5	1/9	1	**1**
**Expert 2**	1	9	7	7	7	**Expert 6**	**1**	9	7	9	9
	1/9	1	1/5	5	5		1/9	**1**	7	1/7	5
	1/7	5	1	5	7		1/7	1/7	**1**	3	9
	1/7	1/5	1/5	1	1		1/9	7	1/3	**1**	1
	1/7	1/5	1/7	1	1		1/9	1/5	1/9	1	**1**
**Expert 3**	**1**	7	9	5	7	**Expert 7**	**1**	9	5	7	9
	1/7	**1**	1/7	5	5		1/9	**1**	1/5	9	5
	1/9	7	**1**	9	7		1/5	5	**1**	7	5
	1/5	1/5	1/9	**1**	1		1/7	1/9	1/7	**1**	1
	1/7	1/5	1/7	1	**1**		1/9	1/5	1/5	1	**1**
**Expert 4**	**1**	7	7	7	9	**Expert 8**	**1**	7	5	7	7
	1/7	**1**	1/7	1/5	1/5		1/7	**1**	1/5	3	5
	1/7	7	**1**	1/7	9		1/5	5	**1**	9	5
	1/7	5	7	**1**	1		1/7	1/3	1/9	**1**	1
	1/9	5	1/9	1	**1**		1/7	1/5	1/5	1	**1**

**Table 17:** RGMM for all the criteria by each expert.

		Expert 1	Expert 2	Expert 3	Expert 4	Expert 5	Expert 6	Expert 7	Expert 8
**RGMM**	**LiFi capacity**	27.7%	60.6%	58.2%	54.1%	61.6%	52.1%	57.9%	55.4%
	**WiFi capacity**	46.4%	10.3%	9.2%	2.7%	9.7%	17.6%	12.5%	10.1%
	**LiFi CDT**	14.5%	21.9%	26.1%	16.7%	22.7%	14.2%	22.7%	26.4%
	**Occurrence rate**	6.8%	3.8%	3.4%	18.8%	3.3%	12.5%	3.4%	3.9%
	**Occupation rate**	4.6%	3.5%	3.1%	7.7%	2.7%	3.6%	5.4%	4.1%

**Table 18:** CM of weighted geometric mean of all participants.

**Consolidated**		**LiFi Capacity**	**WiFi Capacity**	**LiFi CDT**	**Occurrence Rate**	**Occupation Rate**
	**LiFi capacity**		5.089	7.071	6.926	6.223
	**WiFi capacity**	0.196		0.442	2.258	3.599
	**LiFi CDT**	0.141	2.26		3.789	7.297
	**Occurrence rate**	0.144	0.443	0.264		1.275
	**Occupation rate**	0.161	0.278	0.137	0.784	

**Table 19:** AHP weight results.

Matrix	LiFi Capacity	WiFi Capacity	LiFi CDT	Occurrence Rate	Occupation Rate	Normalized Principal Eigenvector 100%	Weights	Values	
**LiFi capacity**	1	5	7	7	6 2/9	59.28%	0.5928	**Consistency Ratio CR:**	**9.1%**
**WiFi capacity**	1/5	1	4/9	2 1/4	3 3/5	11.07%	0.1107	**GCI:**	**0.33**
**LiFi CDT**	1/7	2 1/4	1	3 4/5	7 2/7	19.68%	0.1968	**EVM Check**	**1.2 × 10^−09^**
**Occurrence rate**	1/7	4/9	1/4	1	1 2/7	5.52%	0.0552	**α**	**0.1**
**Occupation rate**	1/6	2/7	1/7	7/9	1	4.45%	0.0445	AHP-S*	**77.8%**
Consistency Ratio (CR), Geometric Consistency Index (GCI), Earned Value Management (EVM), AHP consensus indicator: (AHP-S*), Mean relative Error (MRE), and Lambada (Alonson/Lambada). Note: In this work, we used a linear scale.						SUM = 100%	SUM = 1	**Lambda**	**5.410**
								**Eigenvalue**	**5.40995468**
								**Error:**	**1.0** **× 10^−08^**
								**Iterations**	**7.0** **× 10^+00^**

**Table 20:** VIKOR cost and benefit criteria results.

APs	Benefit Criteria			Cost Criteria	
	LiFi Capacity	WiFi Capacity	LiFi CDT	Occurrence Rate	Occupation Rate
A1	444	41	4.8	2	0.1
A2	435	73	1.9	0.1	0.24
A3	445	35	0.4	3	0.1
A4	446	17	0.8	0.1	0.21
A5	354	59	3.3	13	0.88
A6	428	22	1.7	26	0.55
A7	246	6	0.36	11	0.93
A8	429	14	1.2	28	0.62
A9	439	84	2.5	7	0.34
A10	435	46	1.4	6	0.44
A11	440	32	0.21	8	0.37
F*	446.000	84.000	4.800	0.100	0.100
F-	246.000	6.000	0.210	28.000	0.930

**Table 21:** VIKOR results after using weights of criteria.

Access points	LiFi Capacity	WiFi Capacity	LiFi CDT	Occurrence Rate	Occupation Rate	*S*	*R*	*Q*
Weights	0.5928	0.1107	0.1968	0.0552	0.0445			
A1	0.006	0.061	0.000	0.004	0.000	0.071	0.061	0
A2	0.033	0.016	0.124	0.000	0.008	0.180	0.124	0.121015022
A3	0.003	0.070	0.189	0.006	0.000	0.267	0.189	0.230313159
A4	0.000	0.095	0.172	0.000	0.006	0.272	0.172	0.217331759
A5	0.273	0.035	0.064	0.026	0.042	0.440	0.273	0.406561663
A6	0.053	0.088	0.133	0.051	0.024	0.350	0.133	0.224423969
A7	0.593	0.111	0.190	0.022	0.045	0.960	0.593	1
A8	0.050	0.099	0.154	0.055	0.028	0.387	0.154	0.265688099
A9	0.021	0.000	0.099	0.014	0.013	0.146	0.099	0.077607542
A10	0.033	0.054	0.146	0.012	0.018	0.262	0.146	0.187365961
S*=		**S-**		R*		**R-**		
**0.071**		**0.960**		**0.061**		**0.593**		

**Table 22:** Final VIKOR prioritization results.

Access Points	*S*	*S* Rank	*R*	*R* Rank	*Q*	*Q* Rank
A1	0.071	1	0.061	1	0.0000	1
A2	0.180	3	0.124	3	0.1210	3
A3	0.267	7	0.189	5	0.2303	7
A4	0.272	5	0.172	6	0.2173	5
A5	0.440	10	0.273	10	0.4066	10
A6	0.350	6	0.133	8	0.2244	6
A7	0.960	11	0.593	11	1.0000	11
A8	0.387	9	0.154	9	0.2657	8
A9	0.146	2	0.099	2	0.0776	2
A10	0.262	4	0.146	4	0.1874	4
A11	0.071	8	0.061	7	0.2670	9

**Table 23:** Comparisons of criteria ranking before and after using AHP–VIKOR.

Results		Individual Criteria Ranking (ICR)					AHP–VIKORRanking
Rank Order	Alternative Number and Rule ID	Using LiFi Capacity	Using WiFi Capacity	Using LiFi CDT	Using Occurrence Rate	Using Occupation Rate	Using Weighted Criteria
1st (best)	Alternative number	A4	A9	A1	A2	A1	A1
	Rule ID	474	813	270	1449	270	270
2nd	Alternative number	A3	A2	A5	A4	A3	A9
	Rule ID	370	1449	2781	474	370	813
3rd	Alternative number	A1	A5	A9	A1	A4	A2
	Rule ID	270	2781	813	270	474	1449
4th	Alternative number	A11	A10	A2	A3	A2	A10
	Rule ID	988	1588	1449	370	1449	1588
5th	Alternative number	A9	A1	A6	A10	A9	A4
	Rule ID	813	270	2302	1588	813	474
6th	Alternative number	A2	A3	A10	A9	A11	A6
	Rule ID	1449	370	1588	813	988	2302
7th	Alternative number	A10	A11	A8	A11	A10	A3
	Rule ID	1588	988	2327	988	1588	370
8th	Alternative number	A8	A6	A4	A7	A6	A8
	Rule ID	2327	2302	474	3106	2302	2327
9th	Alternative number	A6	A4	A3	A5	A8	A11
	Rule ID	2302	474	370	2781	2327	988
10th	Alternative number	A5	A8	A7	A6	A5	A5
	Rule ID	2781	2327	3106	2302	2781	2781
11th (worst)	Alternative number	A7	A7	A11	A8	A7	A7
	Rule ID	3106	3106	988	2327	3106	3106

**Table 24:** New weight values for each criterion of five scenarios.

Scenario No.	Scenario Setup		Benefit			Cost	
	Benefit Criteria	Cost Criteria	LiFi Capacity	WiFi Capacity	LiFi CDT	Occurrence Rate	Occupation Rate
Scenario 1	90%	10%	0.9/3	0.9/3	0.9/3	0.1/2	0.1/2
Scenario 2	80%	20%	0.8/3	0.8/3	0.8/3	0.2/2	0.2/2
Scenario 3	70%	30%	0.7/3	0.7/3	0.7/3	0.3/2	0.3/2
Scenario 4	60%	40%	0.6/3	0.6/3	0.6/3	0.4/2	0.4/2
Scenario 5	50%	50%	0.5/3	0.5/3	0.5/3	0.5/2	0.5/2

**Table 25:** New weight values for each criterion of five scenarios.

Weight	Benefit			Cost	
	LiFi Capacity	WiFi Capacity	LiFi CDT	Occurrence Rate	Occupation Rate
Original	0.5928	0.1107	0.1968	0.0552	0.0445
Scenario 1	0.3	0.3	0.3	0.05	0.05
Scenario 2	0.266666667	0.26666667	0.26666667	0.1	0.1
Scenario 3	0.233333333	0.23333333	0.23333333	0.15	0.15
Scenario 4	0.2	0.2	0.2	0.2	0.2
Scenario 5	0.166666667	0.16666667	0.16666667	0.25	0.25

**Table 26:** New priorities for the sensitivity analysis based on new values of *S*, *R*, and *Q*.

Alternatives	Scenario 1				Scenario 2				Scenario 3			
	S	R	Q	Order	S	R	Q	Order	S	R	Q	Order
A1	0.17179	0.165385	0.08452	2	0.156485	0.147009	0.08452	2	0.141181	0.128632	0.050302	2
A2	0.256784	0.189542	0.213016	4	0.237623	0.168482	0.211505	4	0.218462	0.147422	0.181861	3
A3	0.48274	0.287582	0.658991	8	0.434877	0.255628	0.641542	8	0.387014	0.223675	0.620291	8
A4	0.525757	0.261438	0.605597	7	0.474702	0.232389	0.586583	7	0.423647	0.203341	0.557062	6
A5	0.402299	0.138	0.146274	3	0.435495	0.122667	0.18027	3	0.468691	0.140964	0.318792	4
A6	0.5416	0.238462	0.544736	6	0.563116	0.211966	0.572792	6	0.584632	0.18547	0.586246	7
A7	0.95973	0.3	1	11	0.930354	0.266667	1	11	0.900977	0.233333	1	11
A8	0.61135	0.269231	0.683963	9	0.633784	0.239316	0.713418	9	0.656217	0.209402	0.736143	10
A9	0.18765	0.150327	0.04811	1	0.196604	0.133624	0.063967	1	0.205558	0.116921	0.042365	1
A10	0.415931	0.222222	0.414869	5	0.404223	0.197531	0.42001	5	0.392514	0.17284	0.40557	5
A11	0.539423	0.3	0.733287	10	0.51329	0.266667	0.730533	10	0.487157	0.233333	0.727677	9
**Alternatives**	**Scenario 4**				**Scenario 5**							
	**S**	**R**	**Q**	**Order**	**S**		**R**		**Q**		**Order**	
A1	0.125876	0.110256	0.050302	1	0.110572		0.09188		0.025124		1	
A2	0.199302	0.126362	0.180235	3	0.180141		0.105301		0.112973		3	
A3	0.339151	0.191721	0.601513	6	0.291287		0.159768		0.352506		6	
A4	0.372593	0.174292	0.5366	5	0.321538		0.145243		0.329558		5	
A5	0.501887	0.187952	0.691739	7	0.535083		0.23494		0.744874		8	
A6	0.606147	0.185663	0.750176	9	0.627663		0.232079		0.79955		9	
A7	0.8716	0.2	1	11	0.842224		0.25		1		11	
A8	0.678651	0.2	0.87063	10	0.701085		0.25		0.903548		10	
A9	0.214512	0.100218	0.059429	2	0.223465		0.083515		0.07715		2	
A10	0.380806	0.148148	0.411102	4	0.369097		0.123457		0.296629		4	
A11	0.461024	0.2	0.724713	8	0.434892		0.166667		0.471363		7	

**Table 27:** New ranking obtained by the sensitivity analysis for the new scenarios.

Alternatives	Original Rank	S1	S2	S3	S4	S5
A1	1	2	2	2	1	1
A2	3	4	4	3	3	3
A3	7	8	8	8	6	6
A4	5	7	7	6	5	5
A5	10	3	3	4	7	8
A6	6	6	6	7	9	9
A7	11	11	11	11	11	11
A8	8	9	9	10	10	10
A9	2	1	1	1	2	2
A10	4	5	5	5	4	4
A11	9	10	10	9	8	7

## Data Availability

Not applicable.

## Supplementary Materials

The following supporting information can be downloaded at: https://www.mdpi.com/article/10.3390/s23031312/s1.
